# Toward an integrated route to the vernonia allenes and related sesquiterpenoids

**DOI:** 10.3762/bjoc.7.104

**Published:** 2011-07-05

**Authors:** Da Xu, Michael A Drahl, Lawrence J Williams

**Affiliations:** 1 Department of Chemistry and Chemical Biology, Rutgers, The State University of New Jersey, 610 Taylor Road, Piscataway, NJ 08854, USA

**Keywords:** C–C fragmentation, endocyclic allene, natural product, total synthesis

## Abstract

The synthesis of a model endocyclic allene related to the vernonia allenes is described. Fragmentation of a suitable decalin derivative gave the simplified germacrane scaffold. Computational analysis of this and related substrates provides insight into the stereoelectronic requirements of C–C fragmentation. The overall strategy to access these and other sesquiterpenes and the key steps in the present sequence are also discussed.

## Introduction

**Figure 1:**
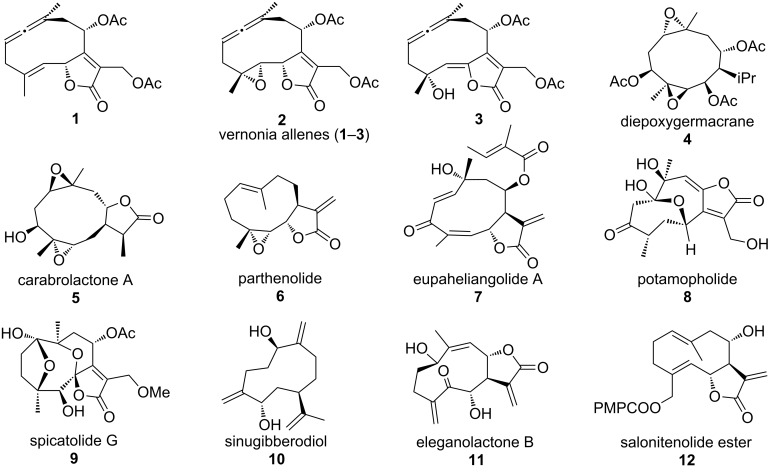
Known natural endocyclic allenes and related germacranes.

Well over 150 allene-containing natural products are known [1–5]. Among these, intriguing complex structures were assigned by Bohlmann and co-workers to isolates from the aerial parts of *Vernonia* species collected mainly from northern Brazil about 30 years ago (**1**–**3**, Figure 1) [6–8]. These bicyclic germacranolides are the only known endocyclic allene-containing natural products. Interestingly, no synthetic studies of these complex natural products have been reported, perhaps in part due to the limited number of routes for cyclic allene construction that are sufficiently mild to effect allene formation in complex settings [9–16].

Our interest in the vernonia allenes was motivated in part by a desire to identify, and to simplify access to, useful bioactive compounds with these sequiterpenoid structures, examples of which are given in Figure 1 (**4**–**12**) [17–23]. Although many of these isolates have not been characterized with regard to their biological function, there is a rich variety of germacranes from terrestrial plants, algae, and insect pheromones that purportedly have anticancer, antifungal, and antibiotic activity, among others. For example, parthenolide (**6**) has anti-inflammatory and anti-hyperalgesic effects and induces apoptosis of human acute myelogenous leukemia stem and progenitor cells [24]; eupaheliangolide A (**7**) is cytotoxic to human oral epidermoid (KB), cervical epitheloid (Hela), and liver (hepa59T/VGH) carcinoma cells [21]; sinugibberodiol (**10**) exhibits beneficial multidrug resistance properties in mammalian tumor cells [25]; eleganolactone B (**11**) inhibits the proliferation of the HL-60 human promyelocytic leukemia cell line in a dose-dependent manner [26], and ester derivatives of salonitenolide **12** show promising antibacterial activity [27].

**Scheme 1:**
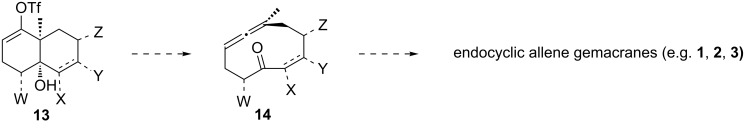
C–C fragmentation strategy to yield endocyclic allenes.

We have initiated a study that aims to integrate the chemical syntheses of compounds in this structure space into a single, late-stage-divergent, route [28]. A pluripotent route that would enable direct access to a broad range of these targets would be useful and would offer advantages over single-target routes, especially in terms of overall step economy. Given the difficulties associated with the stereoselective preparation of endocyclic allenes, and the fact that such structures are largely unexplored, we focused on the vernonia allenes. Moreover, we expected that such allenes contain the coded reactivity to access many sesquiterpenoid variants, especially in light of new methods of transforming allenes to diverse motifs [29–37]. Herein we report our efforts toward this goal with a short synthesis of a model 10-membered endocyclic allene of type **14** (Scheme 1).

## Results and Discussion

**Scheme 2:**
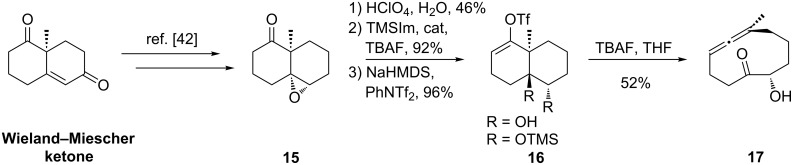
Endocyclic allene **17**.

Advanced intermediates with a high degree of unsaturation have greater potential use in an integrated routing strategy than more highly oxidized products, since not all targets in the group of compounds of interest share identical oxidation states or patterns. Recently, we reported the synthesis of 9- and 10-membered cyclopolyenes [28,38], including a new stereospecific allene synthesis via C–C fragmentation. This transformation appears well-suited for access to the vernonia targets and related compounds [15]. The method relies on suitably functionalized vinyl triflates [39–41]. In general, C–C fragmentation reactions appear to be sensitive to the precise structure of the cyclic system involved, and small, apparently minor, structural changes may severely retard the reaction [38]. For example, in our original disclosure, endocyclic allene **17** was formed from *trans*-decalin derivative **16** by way of the known compound **15** (Scheme 2) [42]. However, the vinyl triflate (**16**, R = OH) resisted fragmentation under standard basic conditions, whereas, the silyl ether (**16**, R = OTMS) underwent smooth fragmentation upon exposure to TBAF.

The ideal orientation for the two bonds that cleave in a C–C fragmentation reaction is antiperiplanar [43–46]. However, to our knowledge, the minimum torsion angle required for fragmentation is not known, and no computational mechanistic study on these reactions has appeared as a guide in this regard. Admittedly, there are distinct differences between fragmentation and substitution; nevertheless, by analogy to S_N_2 reactions, C–C fragmentation substrates with angles significantly less than 180° may fail due to inadequate relative orientation [47–48]. For our purposes, we aimed to identify a scaffold that would readily adopt the ideal, or near-ideal, stereoelectronic arrangement necessary for C–C fragmentation, and yet, would have the potential to accept further structural modification without intrinsic change, should that become necessary or desirable.

Table 1 summarizes the ground state computational modeling of several C–C fragmentation substrates. The Gaussian suite of programs, with the B3LYP functional and the 6-31G(d,p) basis set, was used to generate these data [49–60]. The hydroxy derivatives (R/R’ = OH) and the corresponding alkoxides in vacuum were taken together to approximate the torsion angle of the anion fragmentation precursors. The *trans*-decalin system of entry 1 (c.f. Scheme 2) is highly constrained. The relevant torsion angles are around 155°, thus deviate significantly from 180°. This is true for the mono anions **II** and **III** as well as the diol **I**. Interestingly however, the dianion does not represent a stable structure, and instead gives the endocyclic allene via fragmentation, as observed experimentally. Entry 2 shows a *cis*-decalin system. Low energy conformers were identified for these species [59]. The lowest energy conformer gave the greatest torsion angles (~175°) for both the alcohol **V** and the alkoxide **VI**, and these approach 180°. For comparison purposes, the *cis*-hydrindane derivative in entry 3 was also studied [38]. Analogous to entry 2, this system contains an unsaturated site adjacent to the hydroxy/alkoxide. The torsion angles of the scissile bonds for this entry are approximately 163°. Importantly, this compound is known to undergo base-induced fragmentation to give the (*E*)-alkene in excellent yield [61]. Among the substituted variants we considered, we were intrigued by the *cis*-decalin derivative of entry 4. The ester functionality was taken as a prototype for substitution at this position and represents the potential for both steric and electronic effects. In this case, although both compounds exhibit similar torsion angles (~175°), there appears to be a significant difference between the orientation of the ester relative to the adjacent C–C double bond. For the neutral (R = OH) compound, the ester is coplanar with the double bond, whereas the ester is twisted out of planarity for the alkoxide (R = O^−^). This appears to be an electrostatic influence. The behavior noted in entry 4 was not unique to the *cis*-decalin system and the analogous *cis*-hydrindane exhibits similar behavior (entry 5): The ester group of the hydroxy entry 5 is coplanar with the alkene, whereas the ester is twisted out of planarity for the alkoxide (R = O^−^) [62].

**Table 1:** Computed torsion angles for potential C–C fragmentation substrates.^a^

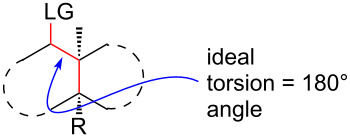			

entry	Substrate	R (R’)	torsion angle

1	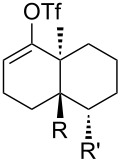	**I**: OH (OH) **II**: OH (O^−^) **III**: O^−^ (OH) **IV**: O^−^ (O^−^)	153° 155° 156° see text
2	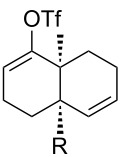	**V**: OH **VI**: O^−^	175° 176°
3	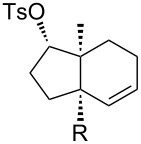	**VII**: OH **VIII**: O^−^	164° 162°
4	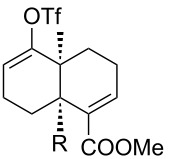	**IX**: OH **X**: O^−^	174° 177°
5	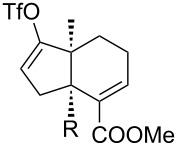	**XI**: OH **XII**: O^−^	171° 170°

^a^DFT: B3LYP functional, 6-31G(d,p) basis set.

In light of the above data, we targeted compound **24** (c.f. **14**, Scheme 1) to extend the C–C fragmentation to *cis*-decalins and to provide an important model for our synthetic studies. This substrate should be able to adopt conformers with the proper orientation for fragmentation and may well tolerate substitution. Given the uncertainties associated with the computational analysis and the precise requirements for fragmentation, it was not clear that even this model compound would undergo C–C fragmentation to give the corresponding endocyclic allene. Consequently, we aimed to prepare **24** by a direct and modular route.

**Scheme 3:**
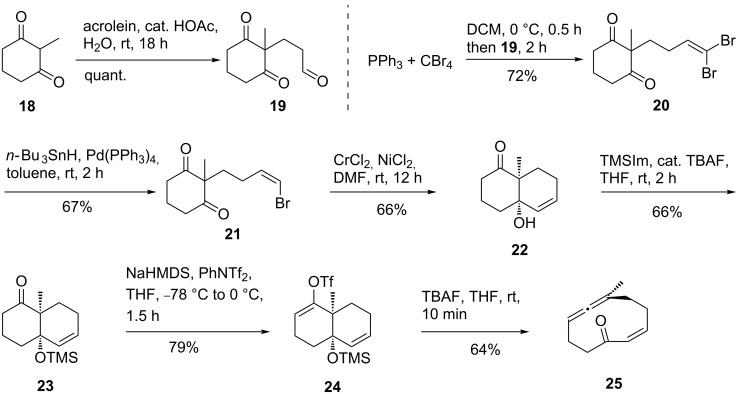
Preparation of endocyclic allene **25**.

**Scheme 4:**
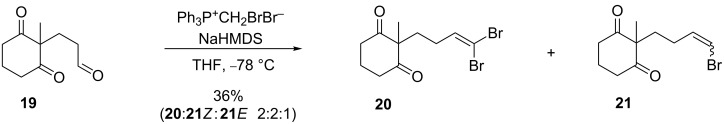
Bromo-olefination products from diketone aldehyde **19**.

Scheme 3 depicts a concise route to **24** and **25**. Beginning with the commercially available diketone **18**, acid promoted Michael addition with acrolein gave aldehyde **19**. A two step procedure, via **20**, was employed to obtain the (*Z*)-bromoolefin **21**. Initially, we examined the direct formation of **21** via bromomethyltriphenylphosphonium bromide (Scheme 4). This reaction was inefficient and gave both the (*E*)- and (*Z*)-bromoolefins as well as dibromoolefin **20**. Bromo group scrambling under basic Wittig reaction conditions is known [63], and the usual procedure for Wittig reagent formation with slow addition of **19** (1 h) gave **21** with the desired olefin geometry but with low selectivity and yield (see below). Rapid addition of **19** (1 min), gave the desired product in 26% yield. The use of HMPA and/or iodomethyltriphenylphosphorane [64–65] was examined and failed to improve the reaction profile, as did varying the ratio of Wittig reagent relative to aldehyde (1–3 equiv), solvent (THF and toluene), and aldehyde concentration (0.035–0.09 M). The yield of the reaction was low under all the conditions examined (13–26%) [66]. The known behavior of bromomethyltriphenylphosphonium bromide under these strongly basic conditions, and the poor solubility of reactive species in THF, account for these results [63].

Alternatively, the *Z*-vinyl bromide **21** was readily obtained via dibromo-olefination of the aldehyde followed by selective removal of the (*E*)-bromide with *n*-Bu_3_SnH and catalytic Pd(0) (**19**→**21**, Scheme 3). A simple sequence was utilized to furnish allene precursor **24**: Thus **21** was converted to bicycle **22** under Nozaki–Hiyama–Kishi conditions [67–68], followed by silyl ether formation (→**23**) [69] and triflation to produce the desired decalin **24**. There is little precedent for Nozaki–Hiyama–Kishi addition to ketones [67–68], but the reaction proceeded without problems in serviceable yield. Brief exposure of **24** to anhydrous fluoride conditions effected clean C–C fragmentation to give the functionalized 10-membered endocyclic allene **25** in good yield.

## Conclusion

These studies demonstrate a concise modular preparation of endocyclic allene **25** under mild reaction conditions. This seven step route gives access to a model system for a synthetic strategy that aims to access the structure space represented by a variety of germacrane natural products, including the vernonia isolates. Computational studies suggest that the diene scaffold may be suitable for further structural modification and adopt the stereoelectronic arrangement necessary for C–C fragmentation. Further studies will be reported in due course.

## Experimental

Preparation of endocyclic allene **25**: In a 25 mL flame-dried flask, vinyl triflate **24** (157 mg, 0.408 mmol, azeotroped with toluene) was suspended in dry THF (13 mL). TBAF (0.4 mL, 0.4 mmol, 1.0 M in THF, stored over molecular sieves for >24 h) was added dropwise. After 10 min, the reaction was quenched by satd. NH_4_Cl (aq) (15 mL) and partitioned against and washed with ethyl acetate (3 × 15 mL). The organic fractions were combined, washed with satd. NaCl (aq) (15 mL), dried (Na_2_SO_4_), filtered, concentrated in vacuo, and purified by FCC (5% ethyl acetate/hexane) to give **25** (43 mg, 64%) as a colorless oil. *R*_f_ 0.08 (1% ethyl acetate/hexane); IR (neat) *v*_max_: 2975, 2903, 2851, 1964, 1693, 1440, 1400 cm^−1^; ^1^H NMR (400 MHz, CDCl_3_) δ 6.25 (dd, *J* = 11.9, 2.2 Hz, 1H), 5.70 (td, *J* = 11.7, 4.2 Hz, 1H), 4.95–4.85 (m, 1H), 2.70–2.57 (m, 2H), 2.42–2.15 (m, 5H), 1.76 (ddt, *J* = 14.7, 12.1, 2.2 Hz, 1H), 1.63 (d, *J* = 2.8 Hz, 3H); ^13^C NMR (101 MHz, CDCl_3_) δ 205.90, 202.06, 140.21, 132.05, 97.82, 89.33, 41.94, 32.71, 26.71, 26.05, 19.93; ESIMS *m*/*z*: [M + Na]^+^calcd for C_11_H_14_NaO, 185.1; found, 185.0.

## Supporting Information

File 1 General experimental methods and analytical data, ^1^H and ^13^C NMR spectra of compounds **18**–**25** and computed structural coordinates for entries 1–5 in Table 1.
