# Supplementary material for: Toward an integrated route to the vernonia allenes and related sesquiterpenoids
Source: Beilstein J Org Chem. 2011 Jul 5;7:937–43. doi: 10.3762/bjoc.7.104 (PMC3135329; doi:10.3762/bjoc.7.104)
Supplement: File 1 — General experimental methods and analytical data, 1H and 13C NMR spectra of compounds 18–25 and computed structural coordinates for entries 1–5 in Table 1. [file Beilstein_J_Org_Chem-07-937-s001.pdf]

Supporting Information

for

Toward an integrated route to the vernonia allenes and related sesquiterpenoids

Da Xu, Michael A. Drahl and Lawrence J. Williams*

Address: Department of Chemistry and Chemical Biology, Rutgers, The State University of New Jersey, 610 Taylor Road, Piscataway, NJ 08854, USA

Email: L. J. Williams - lawjw@rci.rutgers.edu

* Corresponding author

General experimental methods and analytical data, ^{1}H and ^{13}C NMR spectra of compounds **18–25** and computed structural coordinates for entries 1–5 in Table 1

Table of Contents

Experimental details S1–S5
Computational data for Figure 2 S6–S27
^{1}H NMR and ^{13}C NMR spectra of all compounds S29–S43

Experimental Details

General: Starting materials, reagents and solvents were purchased from commercial suppliers (Aldrich, TCI, and Fischer) and used without further purification unless otherwise noted. All reactions were conducted under a dry nitrogen or argon atmosphere unless otherwise mentioned and monitored by thin layer chromatography (thickness 250um with fluorescent indicator, Dynamic Adsorbents Inc.). Crude mixtures were purified by flash column chromatography (FCC) on 32-63 μm silica gel (Dynamic

Adsorbents Inc.). Infrared (FTIR) spectra were recorded on an ATI Mattson Genesis Series FT-Infrared spectrophotometer. Proton nuclear magnetic resonance spectra (^{1}H NMR) were recorded on either a Varian-500 instrument (500 MHz) or a Varian-400 instrument (400 MHz). Carbon nuclear magnetic resonance spectra (^{13}C NMR) were recorded on either Varian-500 instrument (125 MHz) or Varian-400 instrument (100 MHz). Mass spectra were recorded on a Finnigan LCQ-DUO mass spectrometer.

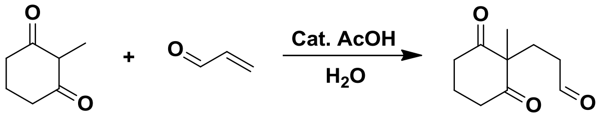

Aldehyde **19**: To a 500 ml round-bottomed flask, **18** (5.00 g, 38.5 mmol) and 60 ml water were added and the mixture was vigorously stirred. Acetic acid (0.11 ml, 2.0 mmol) was added and then acrolein (4.3 ml, 58 mmol) which was added dropwise. After 18 h, the suspension was partitioned against and washed with ethyl acetate (3x60 mL). The organic fractions were combined, washed with satd. NaCl (aq) (60 mL), dried over Na_2SO_4, filtered and concentrated in vacuo to give **19** (7.45 g) as a viscous yellow oil (quantitative crude yield). IR ν_{max}(neat)/cm^{-1} 3435, 2960, 2731, 1722, 1692, 1460; ^{1}H NMR (500 MHz, $CDCl_3$) δ 9.67 (1H, t, J = 1.2 Hz), 2.73 – 2.60 (4H, m), 2.39 – 2.28 (2H, m), 2.13 – 2.07 (2H, m), 2.01 – 1.89 (2H, m), 1.28 (3H, s). ^{13}C NMR (126 MHz, $CDCl_3$) δ 210.03, 201.18, 64.55, 39.54, 38.01, 27.40, 21.78, 17.71.

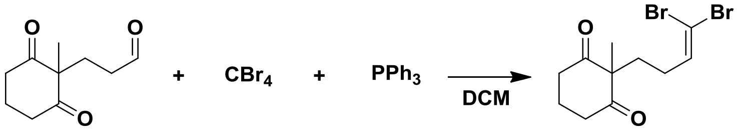

Dibromoalkene **20**: In a 25 ml flame-dried round-bottomed flask, carbon tetrabromide (364 mg, 1.10 mmol) was suspended in dry dichloromethane (3 mL). A solution of triphenylphosphine (576 mg, 2.20 mmol) in dry dichloromethane (3 mL) was added at 0 °C and the reaction mixture stirred for 30 min. Compound **19** (100 mg, 0.549 mmol, azeotroped with toluene) dissolved in dry dichloromethane (3 ml) was then added over 30 minutes. After 1.5 h, the reaction mixture was diluted with hexane (50 ml), filtered, concentrated in vacuo and the resultant oil purified by FCC (15% ethyl acetate in hexane) to give **20** (135 mg, 72%) as a light yellow oil (R_f = 0.80, 50% ethyl acetate in hexane). IR ν_{max}(neat)/cm^{-1} 2962, 1725, 1694, 1456, 1025; ^{1}H NMR (500 MHz, $CDCl_3$) δ 6.37 – 6.25 (1H, m), 2.74 – 2.60 (4H, m), 2.03 – 1.85 (6H, m), 1.27 (3H, s); ^{13}C NMR (126 MHz, $CDCl_3$) δ 210.02, 137.55, 90.24, 64.93, 38.18, 33.82, 28.92, 21.44, 17.78.

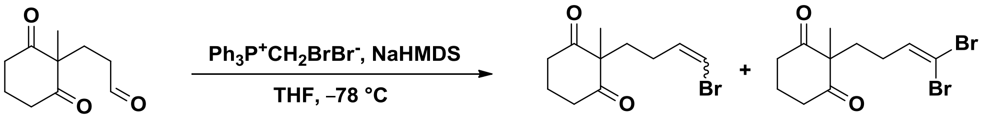

(E/Z)-bromoalkenes **21** and dibromide **20**: (Entry 2, see Table). NaHMDS (0.56 ml, 0.56 mmol, of 1.0 M in THF) was added dropwise to bromomethyltriphenylphosphonium bromide (244 mg, 0.560 mmol) in dry THF (8 mL) at room temperature under vigorous stirring. After 10 min, the dark yellow

suspension was cooled to −78 °C and stirred for an additional 20 min. Separately, **19** (51 mg, 0.28 mmol, azotroped with toluene 3 times and dried under high vacuum) was suspended in dry THF (1 mL) and then added dropwise to the cooled reaction mixture over 1 min. An additional portion of THF (0.5 mL) was used to rinse the flask that had contained **19** and added to the reaction mixture. After 0.5 h, the reaction mixture was diluted with hexane (cooled to −78 °C), warmed to room temperature, filtered, concentrated in vacuo and the resultant oil was purified by FCC (15% ethyl acetate in hexane) to give the monobromo and dibromo products as an inseparable (oil) mixture (22 mg, R_f = 0.80 50% ethyl acetate in hexane, 22% **21,** 8% (*E*)- isomer of **21,** and 5% **20**, the yields are estimated based on ^{1}H NMR of the mixture).

Table 1. Mono bromoolefination of diketone aldehyde **19** under different conditions

Entry	Equiv of Wittig Reagent[a]	Solvent	Reaction Molarity [M]	Reaction Time [h]	Yield[a] [%]	Yield of **21**[a] [%]
1	1.0	THF	0.035	1.0	31	26
2	1.5	THF	0.035	0.5	35	22
3	2.0	THF	0.035	0.5	39	20
4	3.0	THF	0.035	0.5	24	17
5	1.0	Tol.	0.07	1.0	55	25
6	1.2	Tol.	0.02	1.0	38	16
7	1.5	Tol.	0.09	0.5	30	13

[a] Equal amount of phosphonium salt and base were used to generate the Wittig reagent.

[b] The total yields for a mixture of the product were determined from the ^{1}H NMR of the mixture.

[c] The yields of **21** were determined from the ^{1}H NMR of the mixture.

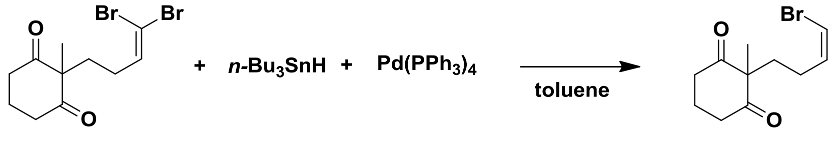

20 **21**

(*Z*)-bromoalkene **21**: Tetrakis[triphenylphosphine]palladium(0) (18 mg, 0.016 mmol) was placed in a flame-dried 10 ml round-bottomed flask under a nitrogen atmosphere (in a glove bag). A solution of dione **20** (133 mg, 0.393 mmol, azeotroped with toluene 3 times) in dry toluene (4 mL) was added with vigorous stirring and then *n*-Bu_3SnH (110 µL, 0.40 mmol). After 2 h, the reaction mixture was diluted with hexanes (4 mL), washed with water (4 mL), satd. NaCl (aq) (4 mL), the organic layer dried over Na_2SO_4, filtered, concentrated in vacuo and the resultant oil purified by FCC (15% ethyl acetate in hexane) to give **21** (135 mg, 67%) as a light yellow oil (R_f = 0.80 50% ethyl acetate in hexane). IR ν_{max}(neat)/cm^{-1} 3084, 2960, 1725, 1694, 1278, 671; ^{1}H NMR (500 MHz, $CDCl_3$) δ 6.20 – 6.09 (1H, m), 6.01 (1H, apparent dt, *J* = 8.2, 6.2 Hz), 2.82 – 2.54 (4H, m), 2.08 – 1.85 (6H, m), 1.26 (3H, s); ^{13}C NMR (126 MHz, $CDCl_3$) δ 210.21, 133.62, 109.16, 65.25, 38.21, 34.88, 25.64, 20.39, 17.87.

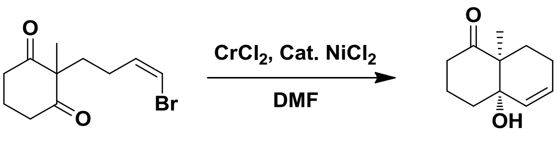

21 **22**

Bicyclic alcohol **22**: To a 100 ml flame-dried round-bottomed flask, chromium(II) chloride (747 mg, 6.08 mmol), nickel(II) chloride (20 mg, 0.15 mmol), and 4Å activated molecular sieves were suspended in 50 mL DMF at 0 °C. Dione **21** (350 mg, 1.35 mmol, azeotroped with toluene 3 times), dissolved in DMF (1 ml) was then added. An additional portion of DMF (1 mL) was used to rinse the flask and added to the reaction mixture, which was then allowed to warm to room temperature. After 12 h, the reaction mixture was diluted with ethyl ether (500 mL), filtered through Florisil, concentrated in vacuo and the residue purified by FCC (20% ethyl acetate in hexane) to give **22** (161 mg, 66%) as a light yellow oil (R_f = 0.53 50% ethyl acetate in hexane). IR ν_{max}(neat)/cm^{-1} 3444, 3016, 2941, 2873, 1695, 1053, 1017; ^{1}H NMR (500 MHz, $CDCl_3$) δ 5.86 – 5.80 (1H, m), 5.53 (1H, apparent dt, *J* = 10.0, 2.2 Hz), 2.42 (1H, ddd, *J* = 15.4, 12.2, 6.4 Hz), 2.27 – 2.15 (2H, m), 2.11 – 2.04 (1H, m), 2.04 – 1.93 (2H, m), 1.88 – 1.80 (1H, m), 1.75 (1H, s), 1.74 – 1.71 (1H, m), 1.53 – 1.43 (2H, m), 1.16 (3H, s); ^{13}C NMR (126 MHz, CD_3CN) δ 213.76, 133.52, 130.54, 72.56, 52.54, 37.30, 34.62, 27.32, 22.77, 20.43, 20.07.

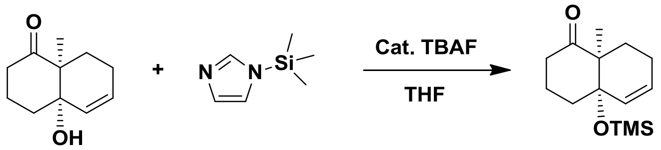

22 23

TMS ether **23**: In a 25 ml pear shaped flask, bicyclic alcohol **22** (150 mg, 0.832 mmol, azeotroped with toluene) was suspended in dry THF (8.3 mL), to which 1-(trimethylsilyl)-1*H*-imidazole (0.13 mL, 0.83 mmol) and one drop of TBAF (1.0 M in THF) were added. After 2 h, the reaction mixture was quenched with satd. NH_4Cl (aq) (8 mL) and partitioned against and then extracted with ethyl acetate (3x8 mL). The organic fractions were combined, washed with satd. NaCl (aq) (8 mL), dried over Na_2SO_4, concentrated in vacuo and the residue purified by FCC (2% ethyl acetate in hexanes) to give **23** (139 mg, 66%) as a colorless oil (R_f =0.76 16% ethyl acetate in hexane). Some unreacted **22** (27 mg, 18%) was also recovered. IR ν_{max}(neat)/cm^{-1} 2950, 1706, 1249, 1100, 1077; ^{1}H NMR (400 MHz, $CDCl_3$) δ 5.85 – 5.77 (1H, m), 5.55 (1H, dt, *J* = 10.0, 2.2 Hz), 2.40 (1H, ddd, *J* = 15.4, 12.7, 6.5 Hz), 2.27 – 2.13 (2H, m), 2.07 (1H, td, *J* = 13.0, 3.8 Hz), 2.00 – 1.85 (2H, m), 1.83 – 1.66 (2H, m), 1.57 (1H, ddd, *J* = 13.3, 10.1, 6.7 Hz), 1.44 (1H, qdd, *J* = 12.7, 4.9, 3.5 Hz), 1.10 (3H, s), 0.13 – -0.02 (9H, m); ^{13}C NMR (101 MHz, $CDCl_3$) δ 214.19, 133.05, 132.52, 74.90, 53.49, 37.56, 35.92, 26.59, 23.30, 21.70, 20.49, 2.54.

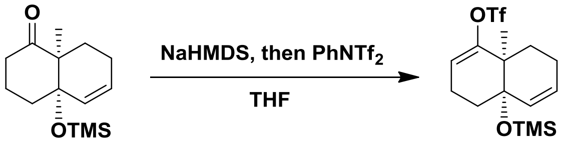

23 24

Vinyl triflate **24**: In a 10 ml pear shaped flask, **23** (138 mg, 0.547 mmol, azeotroped with toluene 3 times) was suspended in dry THF (6 mL) and the mixture was cooled to -78 °C. NaHMDS (0.63 mL, 0.63 mmol, 1.0 M in THF) was added with vigorous stirring. After 30 min, phenyl triflimide (3.0 mL, 0.60 mmol, 0.20 M in THF) was added dropwise and the reaction mixture warmed to 0 °C. After 1 h, the reaction mixture was quenched by satd. NH_4Cl (aq) (10 mL) and partitioned against ethyl acetate (3x10 mL). The organic fractions were combined, washed with satd. NaCl (aq) (10 mL), dried over

Na_2SO_4, concentrated in vacuo, and the residue was purified by FCC (pure hexane) to give **24** (166 mg, 79%) as a colorless oil (R_f = 0.58 1% ethyl acetate in hexane). IR ν_{max}(neat)/cm^{-1} 2952, 1680, 1412, 1248, 1210, 994, 841; ^{1}H NMR (500 MHz, $CDCl_3$) δ 5.84 (1H, dt, J = 9.9, 3.6 Hz), 5.68 – 5.58 (1H, m), 5.57 – 5.45 (1H, m), 2.19 – 2.08 (1H, m), 2.07 – 1.94 (3H, m), 1.88 (1H, ddd, J = 12.8, 10.4, 5.7 Hz), 1.74 (1H, ddd, J = 13.6, 9.4, 6.7 Hz), 1.65 (1H, dt, J = 13.5, 4.4 Hz), 1.60 – 1.52 (1H, m), 1.16 (3H, s), 0.15 – 0.06 (9H, m); ^{13}C NMR (126 MHz, $CDCl_3$) δ 152.88, 130.95, 130.86, 118.58 (q, $J_{C\text{-}F}$ 320Hz), 115.82, 73.86, 43.72, 31.99, 27.69, 22.82, 21.38, 21.31, 2.64.

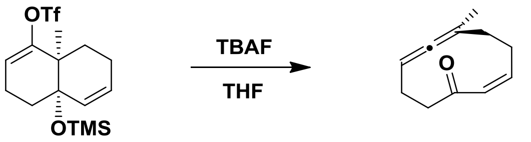

24 25

Endocyclic allene **25**: In a 25 mL flame-dried flask, vinyl triflate **24** (157 mg, 0.408 mmol, azeotroped with toluene 3 times) was suspended in dry THF (13 mL) and TBAF (0.4 mL, 0.4 mmol, 1.0 M in THF, stored over molecular sieves for >24 h) added dropwise. After 10 min, the reaction was quenched with satd. NH_4Cl (aq) (15 mL) and the reaction mixture partitioned against and extracted with ethyl acetate (3x15 mL). The organic fractions were combined, washed with satd. NaCl (aq) (15 mL), dried over Na_2SO_4, concentrated in vacuo and purified by FCC (5% ethyl acetate/hexane) to give **25** (43 mg, 64%) as a colorless oil (R_f = 0.08 1% ethyl acetate/hexane). IR ν_{max}(neat)/cm^{-1} 2975, 2903, 2851, 1964, 1693, 1440, 1400; ^{1}H NMR (400 MHz, $CDCl_3$) δ 6.25 (1H, dd, J = 11.9, 2.2 Hz), 5.70 (1H, td, J = 11.7, 4.2 Hz), 4.95 – 4.85 (1H, m), 2.70 – 2.57 (2H, m), 2.42 – 2.15 (5H, m), 1.76 (1H, ddt, J = 14.7, 12.1, 2.2 Hz), 1.63 (3H, d, J = 2.8 Hz); ^{13}C NMR (101 MHz, CDCl3) δ 205.90, 202.06, 140.21, 132.05, 97.82, 89.33, 41.94, 32.71, 26.71, 26.05, 19.93; m/z (ESI/MS) calculated 185.1 ($C_{11}H_{14}NaO$), observed 185.0 $(M+Na)^+$.

General Computational Procedures: The dihedral angles of all compounds were calculated based on structures which were optimized with the Gaussian 03 suite of programs, utilizing the B3LYP functional with 6-31G(d,p) basis sets.

Computational Data for Table 1:

Entry 1

I:

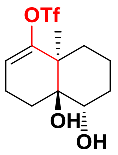

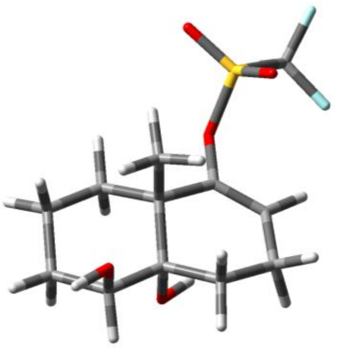

Dihedral angle $\Phi_{((HO)C\text{-}C\text{-}C\text{-}O(Tf))}$ = 152.5°

Atomic Type	Coordinates (Å) X	Y	Z
C	3.953155	-1.289560	-0.880371
C	3.875742	0.027384	-0.092344
C	2.489255	0.691259	-0.242405
C	1.300716	-0.283095	0.114792
C	1.423977	-1.552700	-0.770372
C	2.785452	-2.241423	-0.584128
H	4.593937	0.739856	-0.526553
H	3.958954	-1.037232	-1.945413
H	4.913439	-1.778163	-0.665086
H	0.612377	-2.244604	-0.526877
H	1.302758	-1.274966	-1.822462
H	2.872528	-2.630813	0.436125
H	2.843732	-3.110733	-1.249004
C	0.032278	0.502708	-0.174390
C	2.364841	2.001626	0.550057
H	2.395403	1.787592	1.620302
H	3.229850	2.634152	0.326304
C	1.068114	2.747005	0.174973
H	0.776155	3.431322	0.981145
H	1.241832	3.388119	-0.700332
C	-0.089614	1.831196	-0.131904
H	-1.058126	2.279992	-0.331962
O	2.461007	0.993858	-1.650066

H	1.552393	1.230383	-1.881904
O	4.151389	-0.142674	1.299744
H	5.051150	-0.484797	1.376670
O	-1.100983	-0.287633	-0.518824
C	1.248940	-0.697858	1.611751
H	2.187109	-1.144770	1.935494
H	1.042594	0.157154	2.258432
H	0.443787	-1.424484	1.762802
S	-2.285866	-0.591505	0.576597
O	-2.482285	-2.029584	0.639202
O	-2.131344	0.232606	1.764869
C	-3.702016	0.115624	-0.421140
F	-4.825083	-0.055328	0.273109
F	-3.793989	-0.509069	-1.590407
F	-3.500188	1.420005	-0.624895

HF = -1541.2591036

II:

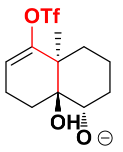

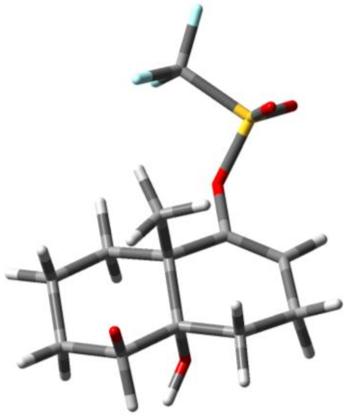

Dihedral angle $\Phi_{((HO)C\text{-}C\text{-}C\text{-}O(Tf))}$ = 155.3°

Atomic	Coordinates (Å)		
Type	X	Y	Z
C	3.514036	-1.863272	-0.612627
C	3.709912	-0.559479	0.254034
C	2.564718	0.457540	-0.164300
C	1.137155	-0.153154	0.062156
C	1.012750	-1.441505	-0.792425
C	2.112527	-2.457416	-0.433536
H	4.628257	-0.043763	-0.235393
H	3.689455	-1.670814	-1.683219
H	4.276247	-2.576263	-0.275619
H	0.021509	-1.885300	-0.639346

H	1.092735	-1.174130	-1.851532
H	2.000166	-2.770862	0.610421
H	1.982207	-3.358884	-1.050403
C	0.151056	0.918547	-0.340917
C	2.692386	1.786929	0.584099
H	2.561748	1.605870	1.653074
H	3.716745	2.160176	0.457328
C	1.692188	2.825469	0.040595
H	1.541731	3.643311	0.760184
H	2.103878	3.288824	-0.867000
C	0.355587	2.233019	-0.328144
H	-0.443941	2.906977	-0.628347
O	3.817919	-0.777369	1.551433
O	-1.146470	0.428769	-0.776826
C	0.904224	-0.501067	1.563596
H	1.835118	-0.934022	1.953882
H	0.671514	0.396502	2.143200
H	0.056243	-1.188002	1.667624
S	-2.503301	0.765244	0.022342
O	-3.403580	1.522396	-0.840839
O	-2.268507	1.170590	1.400963
C	-3.180513	-0.975037	0.060440
F	-4.393261	-0.939095	0.629332
F	-3.296414	-1.454873	-1.178767
F	-2.392221	-1.778802	0.773175
O	2.680091	0.741389	-1.582612
H	3.624488	0.601771	-1.760777

HF = -1540.6603569

III:

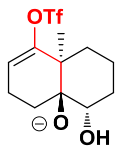

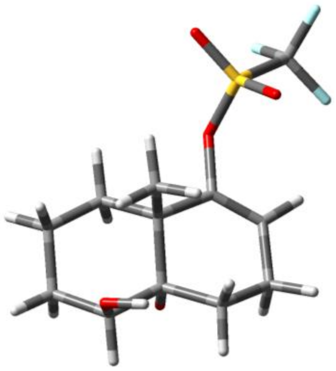

Dihedral angle $\Phi_{((O^-)C\text{-}C\text{-}C\text{-}O(Tf))}$ = 156.0°

Atomic		Coordinates (Å)	
Type	X	Y	Z
C	4.066694	-1.172988	-0.861928

C	3.919447	0.062727	0.029631
C	2.548430	0.755057	-0.276490
C	1.329664	-0.305908	0.057802
C	1.524114	-1.449677	-0.963606
C	2.881596	-2.149093	-0.763263
H	4.674682	0.803346	-0.280955
H	4.120415	-0.785887	-1.882647
H	5.008989	-1.688716	-0.629530
H	0.709060	-2.182078	-0.891649
H	1.502037	-0.987998	-1.954932
H	2.902354	-2.663549	0.205724
H	2.996812	-2.935340	-1.523475
C	0.076868	0.482397	-0.179736
C	2.303397	2.010802	0.633801
H	2.140751	1.784554	1.700873
H	3.176706	2.673719	0.561866
C	1.076896	2.730121	0.056727
H	0.775705	3.597245	0.660893
H	1.371942	3.075572	-0.943140
C	-0.092121	1.799466	-0.130885
H	-1.078625	2.222006	-0.298703
O	2.543083	1.101258	-1.561424
O	4.195145	-0.325033	1.401388
H	3.827296	0.379693	1.949698
O	-1.114857	-0.335640	-0.493726
C	1.243726	-0.880591	1.497892
H	2.179316	-1.355649	1.792763
H	1.015980	-0.097129	2.227286
H	0.442207	-1.629743	1.571427
S	-2.286976	-0.614784	0.556863
O	-2.577532	-2.043549	0.592253
O	-2.166393	0.163567	1.783100
C	-3.693119	0.146659	-0.407163
F	-4.830807	-0.027557	0.280768
F	-3.818652	-0.434853	-1.601629
F	-3.497555	1.458836	-0.579664

HF=-1540.6634949

Entry 2

V: 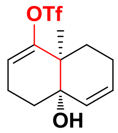

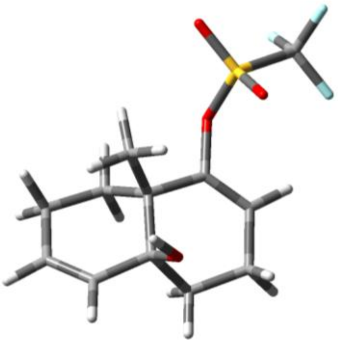

Dihedral angle $\Phi_{((HO)C\text{-}C\text{-}C\text{-}O(Tf))}$ = 175.4°

Atomic Type	X	Coordinates (Å) Y	Z
C	0.165719	1.738533	-0.493703
C	0.280211	0.423895	-0.324132
C	1.534678	-0.367612	-0.011120
C	2.701722	0.630261	0.342628
O	-0.864633	-0.403609	-0.556295
C	-3.444894	0.177602	-0.393468
F	-4.558077	0.051542	0.326309
F	-3.199888	1.473862	-0.604969
F	-3.589560	-0.443120	-1.560242
S	-2.036749	-0.590547	0.571003
O	-2.330426	-2.010732	0.668024
O	-1.795465	0.245113	1.736925
C	1.264194	-1.315931	1.181322
C	1.930302	-1.198486	-1.266198
C	3.211668	-2.018013	-1.063518
C	4.283276	-1.218896	-0.373838
C	4.049882	-0.052758	0.234389
O	2.552451	1.150492	1.673585
C	2.662547	1.886526	-0.547982
C	1.348506	2.656170	-0.376872
H	-0.809568	2.160227	-0.714528
H	0.838791	-0.771098	2.027217
H	2.186700	-1.804885	1.507574
H	0.558737	-2.101603	0.899611
H	2.075389	-0.513865	-2.109203
H	1.096371	-1.853919	-1.536620
H	3.006303	-2.929975	-0.484018
H	3.578810	-2.372434	-2.035235
H	5.291766	-1.629311	-0.371891
H	4.861236	0.490528	0.716128

H	2.702741	0.426963	2.295755
H	2.788563	1.588242	-1.593379
H	3.514454	2.521471	-0.285323
H	1.328408	3.148707	0.603317
H	1.274741	3.451295	-1.128309

HF = -1464.818396

VI:

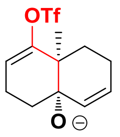

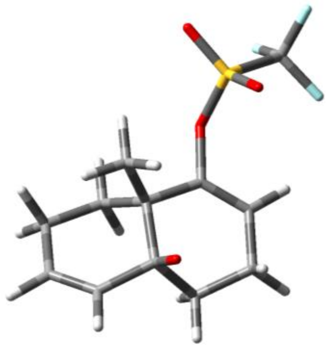

Dihedral angle $\Phi_{((O^{-})C\text{-}C\text{-}C\text{-}O(Tf))}$ = 176.2°

Atomic Type	Coordinates (Å) X	Y	Z
C	0.191015	1.732476	-0.400734
C	0.337014	0.420671	-0.232781
C	1.578440	-0.372760	0.049445
C	2.784123	0.692376	0.378544
O	-0.863518	-0.414896	-0.460555
C	-3.429373	0.164032	-0.441996
F	-3.542748	-0.485680	-1.603769
F	-4.584232	0.052393	0.229307
F	-3.207150	1.460343	-0.691395
S	-2.056993	-0.564277	0.596253
O	-1.921111	0.303584	1.756785
O	-2.401252	-1.975293	0.729846
C	1.375107	-1.225173	1.314954
C	1.911706	-1.253246	-1.184795
C	3.200148	-2.070914	-1.010297
C	4.314817	-1.241953	-0.424338
C	4.115898	-0.034491	0.118418
O	2.742577	1.135561	1.630794
C	2.653107	1.888451	-0.642213
C	1.377308	2.646374	-0.274195
H	-0.794370	2.142169	-0.602670
H	1.232247	-0.544729	2.155903
H	2.274823	-1.809962	1.524438
H	0.532277	-1.920483	1.215570

H	2.029785	-0.597682	-2.056641
H	1.073270	-1.926433	-1.411893
H	3.007660	-2.951081	-0.375539
H	3.502366	-2.484295	-1.985797
H	5.313622	-1.685785	-0.431977
H	4.956374	0.510995	0.549793
H	2.630066	1.574004	-1.696997
H	3.528606	2.534661	-0.501764
H	1.498666	2.920871	0.785034
H	1.224243	3.555991	-0.869821

HF=-1464.2166481

Entry 2(0.16 Kcal lower energy conformer)

V:

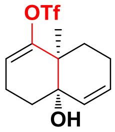

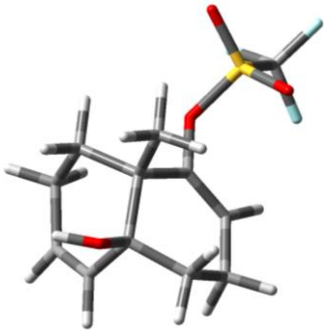

Dihedral angle $\Phi_{((HO)C\text{-}C\text{-}C\text{-}O(Tf))}$ = 163.4°

Atomic Type	Coordinates (Å) X	Y	Z
C	0.452196	1.420981	-0.855694
C	0.435583	0.285479	-0.162139
C	1.570952	-0.340095	0.627000
C	2.915817	0.373886	0.207995
O	-0.758574	-0.501649	-0.164529
C	-3.294577	0.045503	-0.605335
F	-3.371285	-1.102422	-1.271687
F	-4.477022	0.334604	-0.065600
F	-2.937015	1.024387	-1.440688
S	-2.040062	-0.098290	0.775211
O	-1.875214	1.222438	1.361342
O	-2.406751	-1.264601	1.561225
C	1.296659	-0.178434	2.139319
C	1.680176	-1.854391	0.305349
C	1.936748	-2.139880	-1.176986
C	3.029898	-1.264110	-1.722498
C	3.453554	-0.156623	-1.106669

C	2.719964	1.896295	0.119053
C	1.677021	2.286127	-0.934157
H	-0.445788	1.735021	-1.378649
H	1.148504	0.867467	2.417276
H	2.137223	-0.568899	2.715542
H	0.397626	-0.733374	2.424376
H	0.775419	-2.366797	0.642628
H	2.508359	-2.258567	0.902063
H	2.200696	-3.195800	-1.316182
H	1.016646	-1.987687	-1.759787
H	3.489273	-1.559183	-2.664554
H	4.268062	0.426727	-1.533848
H	3.688747	2.353521	-0.105559
H	2.417166	2.264728	1.104327
H	1.390007	3.337095	-0.808914
H	2.103341	2.209862	-1.944725
O	3.925763	0.192291	1.215678
H	4.287877	-0.696852	1.103258

HF = -1464.8186512

VI:

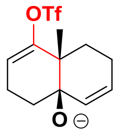

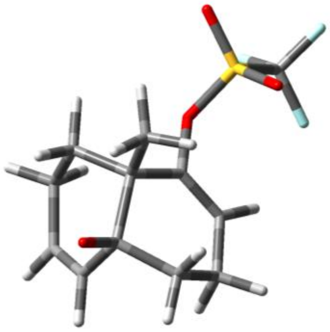

Dihedral angle $\Phi_{((O^{-})C\text{-}C\text{-}C\text{-}O(Tf))}$ = 163.6°

Atomic Type	Coordinates (Å) X	Y	Z
C	0.472268	1.515266	-0.603002
C	0.491973	0.294501	-0.077598
C	1.602218	-0.432079	0.598100
C	3.044085	0.344879	0.332633
O	-0.765594	-0.496078	-0.235074
C	-3.276339	0.201632	-0.581893
F	-4.473051	0.362815	0.000373
F	-2.922634	1.360063	-1.152742
F	-3.380822	-0.732920	-1.529863
S	-2.034356	-0.306992	0.718145
O	-2.489576	-1.610111	1.188490

O	-1.912110	0.827425	1.624581
C	1.380585	-0.493241	2.120636
C	1.780909	-1.868963	0.061749
C	1.995077	-1.932112	-1.455111
C	3.077139	-0.973820	-1.886221
C	3.492863	0.032137	-1.107456
O	3.974019	-0.050592	1.187269
C	2.750139	1.885004	0.415662
C	1.693182	2.396313	-0.574877
H	-0.438225	1.887760	-1.064282
H	1.020083	0.460179	2.519142
H	2.368234	-0.702317	2.547703
H	0.661418	-1.272689	2.404532
H	2.681999	-2.240345	0.563964
H	0.934402	-2.502445	0.356367
H	2.266228	-2.959156	-1.743830
H	1.049267	-1.722563	-1.982926
H	3.549451	-1.155810	-2.854518
H	4.322776	0.656673	-1.446752
H	2.442322	2.104682	1.445293
H	3.704665	2.402207	0.257439
H	1.382278	3.425648	-0.339041
H	2.114853	2.438172	-1.591186

HF = -1464.2178719

Entry 3

VII:

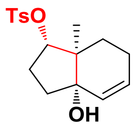

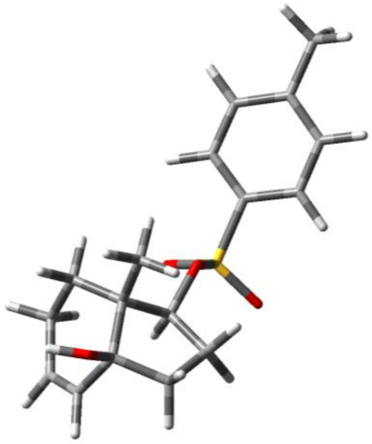

Dihedral angle $\Phi_{((HO)\text{-}C\text{-}C\text{-}C\text{-}O(Tf))}$ = 163.9°

Atomic		Coordinates (Å)	
Type	X	Y	Z
O	-0.009055	0.008868	0.015554

C	0.019665	-0.018131	3.903182
C	2.647406	0.014214	1.010752
C	0.763323	-2.336722	0.348390
C	1.757846	-2.960295	1.359261
C	0.325088	-1.018808	1.001664
C	1.512386	-0.558859	1.875837
C	1.935183	-1.930651	2.510215
C	1.114318	0.473477	2.947246
C	0.247069	-1.441221	4.333219
C	1.079727	-2.281778	3.709222
O	3.313709	-1.980261	2.905578
H	-0.024142	0.631785	4.786493
H	-0.969775	0.072021	3.431980
H	2.974041	-0.682631	0.234065
H	2.321422	0.935505	0.521161
H	3.517292	0.232618	1.633960
H	1.253358	-2.114078	-0.603450
H	-0.094442	-2.975496	0.127204
H	1.415320	-3.924067	1.746452
H	2.737670	-3.127762	0.904794
H	-0.551915	-1.180763	1.632191
H	0.799766	1.403397	2.460763
H	2.015259	0.717565	3.527389
H	-0.319464	-1.798013	5.192111
H	1.203691	-3.299341	4.077211
H	3.383289	-1.523141	3.755129
O	-2.213068	0.773240	0.975178
S	-1.609343	0.244439	-0.251394
O	-2.169889	-0.954036	-0.876839
C	-1.468973	1.545522	-1.459831
C	-1.453929	2.875021	-1.037126
C	-1.388718	1.212841	-2.812282
C	-1.344987	3.882883	-1.991694
H	-1.535506	3.108234	0.018572
C	-1.280374	2.235420	-3.750999
H	-1.419373	0.172598	-3.116518
C	-1.256888	3.581586	-3.358546
H	-1.331386	4.920894	-1.671072
H	-1.215801	1.984921	-4.806242
C	-1.172097	4.682788	-4.386952
H	-0.627305	4.357488	-5.277640
H	-2.173484	4.991397	-4.711422
H	-0.672728	5.568772	-3.984922

HF = -1360.0986911

VIII:

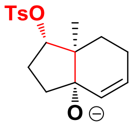

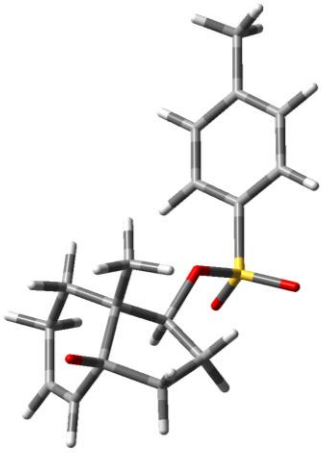

Dihedral angle $\Phi_{((O^{-})C\text{-}C\text{-}C\text{-}O(Tf))}$ = 162.3°

Atomic Type	Coordinates (Å) X	Y	Z
O	-0.011197	0.549685	0.265487
C	2.383853	-2.052078	-1.397424
C	1.779598	-0.639773	2.190224
C	2.157623	1.846162	0.338228
C	3.586853	1.434759	0.774343
C	1.472230	0.526422	-0.057124
C	2.152553	-0.592295	0.705549
C	3.731192	-0.141000	0.616169
C	2.008072	-1.989580	0.090674
C	3.679424	-1.327143	-1.671930
C	4.224412	-0.459934	-0.807301
O	4.525472	-0.727209	1.493578
H	2.470673	-3.104683	-1.708292
H	1.567995	-1.640514	-2.017576
H	1.664905	0.359143	2.625500
H	0.843200	-1.184953	2.363271
H	2.616952	-1.131481	2.698375
H	1.600380	2.304584	1.161474
H	2.132343	2.559876	-0.492916
H	4.367303	1.966700	0.215868
H	3.752550	1.639353	1.836866
H	1.525655	0.362584	-1.133089
H	0.995714	-2.392640	0.245482
H	2.707535	-2.623114	0.652721
H	4.184689	-1.560013	-2.612350
H	5.190661	-0.009787	-1.045372
O	-0.695280	1.021607	-2.119623

S	-1.017721	1.323376	-0.718468
O	-1.185523	2.725978	-0.316871
C	-2.520673	0.447344	-0.281366
C	-2.772492	-0.806204	-0.840855
C	-3.420178	1.024273	0.613063
C	-3.936954	-1.484718	-0.491487
H	-2.062786	-1.234887	-1.539916
C	-4.582065	0.332190	0.951985
H	-3.202480	2.001971	1.028323
C	-4.859032	-0.928280	0.407146
H	-4.133809	-2.463697	-0.921640
H	-5.284305	0.778059	1.652352
C	-6.134353	-1.658831	0.755926
H	-6.488025	-1.390971	1.756223
H	-6.938591	-1.414127	0.050002
H	-5.995176	-2.743813	0.724684

HF = -1359.4909522

Entry 4

IX:

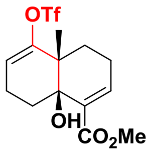

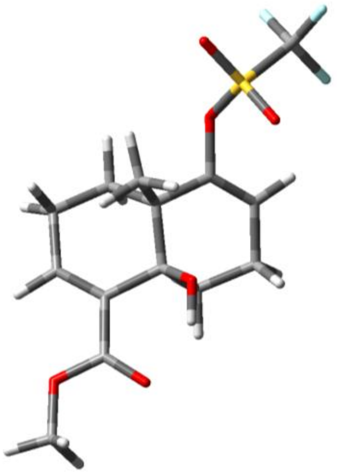

Dihedral angle $\Phi_{((HO)C\text{-}C\text{-}C\text{-}O(Tf))}$ = 174.4°

Atomic Type	Coordinates (Å) X	Y	Z
C	-0.486232	-2.389065	-0.978028
C	0.759435	-1.562482	-0.845200
C	0.711314	-0.285480	-0.476702
C	-0.516216	0.548891	-0.159876
C	-1.761276	-0.400371	-0.047062
C	-1.725418	-1.502321	-1.136759
O	1.919143	0.479117	-0.472802
C	4.443927	-0.254738	-0.235998

F	5.496857	-0.348547	0.574330
F	4.716717	0.581646	-1.233380
F	4.159253	-1.462386	-0.731807
S	2.995177	0.386851	0.759557
O	3.336153	1.743496	1.154075
O	2.626402	-0.644448	1.715517
C	-0.304041	1.304801	1.173656
C	-0.723422	1.560410	-1.320371
C	-1.958821	2.441387	-1.117855
C	-3.141397	1.659894	-0.638302
C	-3.068372	0.405444	-0.160025
C	-4.305094	-0.288992	0.293975
O	-4.324663	-1.404081	0.803709
O	-5.435314	0.414468	0.089337
C	-6.648899	-0.215956	0.534568
H	-0.592159	-3.020890	-0.087137
H	-0.393521	-3.067092	-1.834592
H	1.723265	-2.021140	-1.041424
H	-1.731535	-1.041889	-2.131248
H	-2.634481	-2.105997	-1.057069
H	-0.020680	0.612252	1.966116
H	0.474692	2.064474	1.065029
H	-1.224501	1.801921	1.487901
H	0.173628	2.179021	-1.417248
H	-0.826457	1.010234	-2.262104
H	-2.216884	2.957972	-2.051472
H	-1.758255	3.244965	-0.393922
H	-4.109793	2.151571	-0.665105
H	-6.609655	-0.410242	1.608760
H	-6.805190	-1.161021	0.009565
H	-7.445187	0.489720	0.300349
O	-1.675038	-1.017278	1.235622
H	-2.507969	-1.508624	1.348617

HF = -1692.7032593

X:

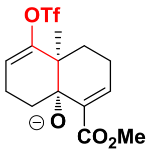

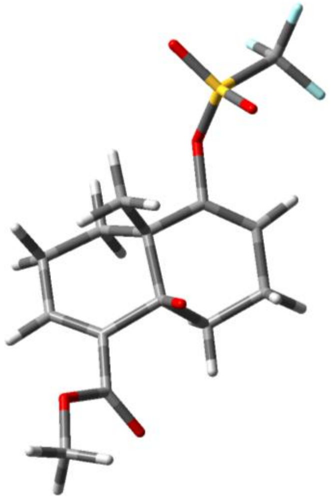

Dihedral angle $\Phi_{((O^{-})C\text{-}C\text{-}C\text{-}O(Tf))}$ = 176.8°

Atomic	Coordinates (Å)		
Type	X	Y	Z
C	0.493142	2.513527	-0.544625
C	-0.752513	1.672966	-0.527712
C	-0.665706	0.353123	-0.387503
C	0.555263	-0.510002	-0.239638
C	1.846572	0.478841	-0.063875
C	1.673069	1.684865	-1.053110
O	-1.921168	-0.413821	-0.480707
C	-4.428080	0.312163	-0.148831
F	-5.505369	0.238108	0.644522
F	-4.701087	-0.297027	-1.305497
F	-4.164966	1.601990	-0.389638
S	-2.989820	-0.514543	0.711583
O	-3.396076	-1.906522	0.864868
O	-2.662013	0.320733	1.857111
C	0.437745	-1.349544	1.046390
C	0.702058	-1.409569	-1.495023
C	1.931638	-2.325116	-1.424483
C	3.148787	-1.602437	-0.915698
C	3.104016	-0.352591	-0.427135
O	2.005070	0.892935	1.193367
C	4.367870	0.303452	0.024336
O	4.777262	1.397656	-0.314397
O	5.118953	-0.511220	0.830665
C	6.345681	0.065776	1.273188
H	0.744754	2.796519	0.488125
H	0.326902	3.426001	-1.131840
H	-1.728896	2.141054	-0.613177

H	1.501486	1.371838	-2.094603
H	2.596411	2.267827	-1.020914
H	0.382359	-0.667801	1.895768
H	-0.430211	-2.019847	1.020388
H	1.334980	-1.957655	1.185313
H	-0.202264	-2.015060	-1.641759
H	0.792561	-0.763274	-2.376713
H	2.132015	-2.752237	-2.419703
H	1.724076	-3.192680	-0.778471
H	4.091708	-2.149981	-0.928385
H	6.162619	0.984896	1.837482
H	7.001566	0.308172	0.430034
H	6.816361	-0.683534	1.913699

HF = -1692.1008936

Entry 4(8.55 Kcal higher energy conformer)

IX:

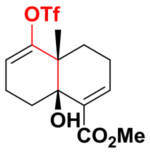

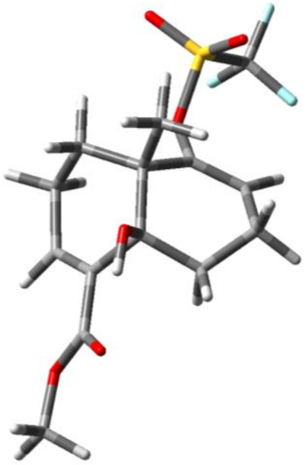

Dihedral angle $\Phi_{((HO)C\text{-}C\text{-}C\text{-}O(Tf))}$ = 141.4°

Atomic Type	Coordinates (Å) X	Y	Z
C	0.623461	1.981985	-1.750082
C	-0.524922	1.073609	-1.414082
C	-0.519566	0.460170	-0.233856
C	0.506846	0.711836	0.865400
C	1.959896	0.851837	0.225316
C	1.934962	1.346996	-1.266409
O	-1.558099	-0.463471	0.078593
C	-4.025900	-0.702611	-0.839555
F	-5.316743	-0.473375	-0.608789
F	-3.805865	-2.011687	-0.912321
F	-3.670798	-0.124971	-1.989863

S	-3.041574	0.035116	0.571067
O	-3.383912	-0.718795	1.765216
O	-3.172902	1.481404	0.489102
C	0.102805	2.035215	1.573321
C	0.496644	-0.402921	1.934644
C	0.932718	-1.766488	1.399157
C	2.253847	-1.649371	0.708270
C	2.729074	-0.484250	0.240210
O	2.654273	1.792700	1.044713
C	4.129627	-0.405650	-0.257441
O	4.767576	0.637830	-0.337815
O	4.661965	-1.595338	-0.597046
C	6.043482	-1.565103	-0.997054
H	0.474784	2.970435	-1.296164
H	0.675673	2.148039	-2.830890
H	-1.333541	0.910120	-2.118111
H	2.131022	0.492573	-1.923671
H	2.767260	2.042466	-1.395249
H	0.151496	2.893730	0.901996
H	-0.923731	1.954688	1.940971
H	0.769086	2.231390	2.413202
H	1.192271	-0.095305	2.722667
H	-0.495777	-0.470849	2.388080
H	0.179577	-2.180457	0.714493
H	1.015788	-2.489743	2.219452
H	2.874232	-2.537416	0.625525
H	6.305826	-2.599901	-1.214646
H	6.668625	-1.169480	-0.193223
H	6.171807	-0.940969	-1.884227
H	3.572394	1.782971	0.722325

HF = -1692.6896301

X:

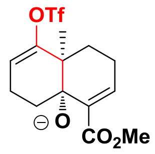

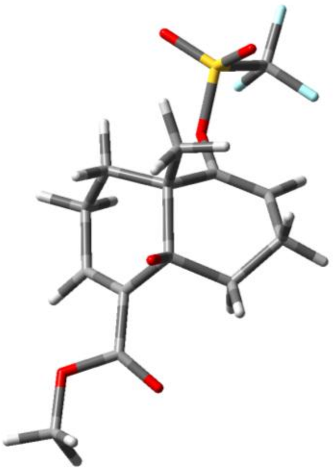

Dihedral angle $\Phi_{((O^{-})C\text{-}C\text{-}C\text{-}O(Tf))}$ = 138.3°

Atomic Type	Coordinates (Å) X	Y	Z
C	0.534884	2.671645	-0.425951
C	-0.614111	1.714870	-0.586969
C	-0.546717	0.556339	0.066095
C	0.507409	0.165984	1.048929
C	2.062555	0.716804	0.546995
C	1.869286	1.909968	-0.488566
O	-1.614613	-0.420701	-0.215221
C	-4.114808	-0.144994	-0.973512
F	-5.397951	-0.165620	-0.588281
F	-3.913020	-1.123893	-1.857895
F	-3.863293	1.030755	-1.559902
S	-3.033980	-0.379030	0.532061
O	-3.354804	-1.710230	1.032553
O	-3.213409	0.809244	1.355421
C	0.234968	0.870669	2.396424
C	0.568725	-1.346475	1.317143
C	1.009024	-2.178270	0.110051
C	2.356908	-1.713205	-0.359708
C	2.779768	-0.447231	-0.202076
O	2.804682	1.012435	1.593532
C	4.169841	-0.114810	-0.633362
O	4.556899	0.909990	-1.161876
O	5.045318	-1.155352	-0.416600
C	6.376887	-0.905459	-0.856776
H	0.452588	3.203110	0.533141
H	0.494416	3.441507	-1.207492
H	-1.462852	1.949391	-1.221522
H	1.982098	1.545361	-1.516348

H	2.703037	2.587135	-0.295327
H	-0.103535	1.902029	2.264410
H	-0.533311	0.341160	2.974449
H	1.193807	0.889970	2.925178
H	1.307183	-1.483614	2.115814
H	-0.395061	-1.709140	1.697232
H	0.256889	-2.118930	-0.691249
H	1.060793	-3.240490	0.387565
H	3.030271	-2.455579	-0.784858
H	6.950670	-1.799604	-0.601857
H	6.798546	-0.029284	-0.355083
H	6.416222	-0.727820	-1.937105

HF = -1692.0872292

Entry 5

XI:

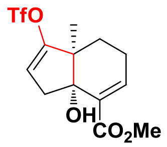

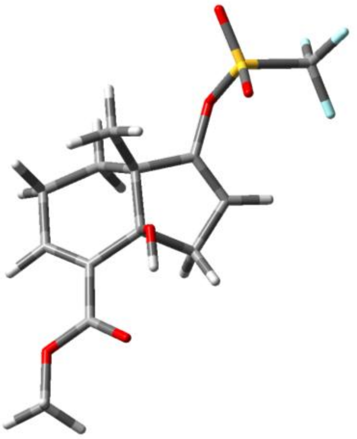

Dihedral angle $\Phi_{((HO)C\text{-}C\text{-}C\text{-}O(Tf))}$ = 171.3°

Atomic Type	Coordinates (Å) X	Y	Z
C	0.314432	-1.088452	-0.999010
C	0.633724	0.080986	-0.452553
C	-0.536360	0.892759	0.066824
C	-1.639750	-0.226985	0.130739
O	1.909986	0.671843	-0.487625
C	4.189120	-0.699681	-0.450371
F	4.599550	0.040170	-1.475827
F	5.235543	-1.081951	0.278645
F	3.548354	-1.779233	-0.906394
S	3.046762	0.301843	0.643823
O	2.513239	-0.593688	1.655436
O	3.742503	1.536476	0.957707

C	-0.260464	1.549061	1.427940
C	-0.906523	1.959314	-0.999124
C	-2.270145	2.604233	-0.737612
C	-3.325191	1.572344	-0.477608
C	-3.051159	0.303845	-0.127991
C	-1.171571	-1.305912	-0.906547
H	1.010082	-1.774200	-1.466607
H	0.067898	0.809434	2.157471
H	-1.161142	2.025746	1.823508
H	0.513744	2.316513	1.322809
H	-0.932473	1.482689	-1.986398
H	-0.118705	2.718891	-1.037401
H	-2.227870	3.295718	0.116602
H	-2.567987	3.222687	-1.593801
H	-4.364255	1.873316	-0.578447
H	-1.443845	-2.306266	-0.557250
H	-1.646580	-1.167738	-1.888049
C	-4.148447	-0.673214	0.104366
O	-3.975239	-1.800888	0.551576
O	-5.371006	-0.217262	-0.229617
C	-6.459514	-1.125100	0.014637
H	-6.518149	-1.378153	1.075748
H	-7.356740	-0.595698	-0.304179
H	-6.329232	-2.043973	-0.561335
O	-1.577993	-0.784706	1.438491
H	-2.264799	-1.472719	1.458469

HF = -1695.37974860

XII:

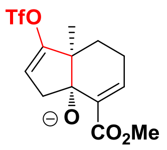

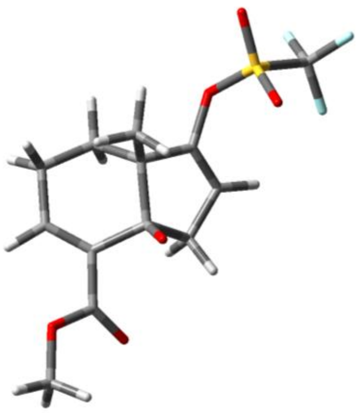

Dihedral angle $\Phi_{((O^{-})C\text{-}C\text{-}C\text{-}O(Tf))}$ = 169.6°

Center Number	Atomic Number	Atomic Type	Coordinates (Å) X	Y	Z
		C	0.352606	-1.190884	-0.774519

C	0.604928	0.054425	-0.378992
C	-0.566451	0.902577	0.005052
C	-1.710629	-0.279852	0.194529
O	1.921236	0.652456	-0.430820
C	4.201391	-0.712282	-0.382752
F	4.532423	-0.080184	-1.511936
F	5.317070	-0.966518	0.314269
F	3.615929	-1.873996	-0.690378
S	3.071087	0.352706	0.658821
O	2.620371	-0.479907	1.762043
O	3.812137	1.589627	0.871444
C	-0.361004	1.634958	1.331700
C	-0.914611	1.863278	-1.157678
C	-2.287859	2.525467	-0.994234
C	-3.356624	1.512524	-0.685293
C	-3.074055	0.253199	-0.306809
C	-1.120079	-1.462834	-0.706027
H	1.109018	-1.899616	-1.098047
H	-0.304996	0.887323	2.125240
H	-1.217924	2.276461	1.558660
H	0.539721	2.262996	1.314931
H	-0.921735	1.288550	-2.093290
H	-0.135283	2.631969	-1.268615
H	-2.255330	3.291768	-0.203461
H	-2.544355	3.074985	-1.913554
H	-4.393102	1.838804	-0.764105
H	-1.363574	-2.406219	-0.207800
H	-1.555985	-1.500300	-1.715786
C	-4.182199	-0.721581	-0.109819
O	-4.172812	-1.894447	-0.432370
O	-5.311121	-0.149460	0.424620
C	-6.409671	-1.043895	0.577752
H	-6.145455	-1.881300	1.230471
H	-7.215955	-0.457127	1.024146
H	-6.730595	-1.453602	-0.386269
O	-1.900449	-0.633556	1.451495

HF = -1652.7776413

Entry 5(1.04 Kcal lower energy conformer)

XI:

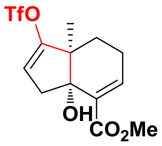

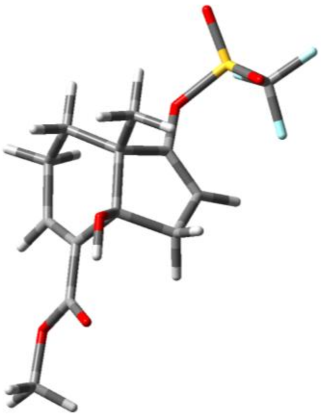

Dihedral angle $\Phi_{((HO)C\text{-}C\text{-}C\text{-}O(Tf))}$ = 163.7°

Atomic Type	Coordinates (Å) X	Y	Z
C	1.269058	0.282842	2.410785
C	2.458016	-0.283676	1.697992
C	2.731718	-0.034833	0.406829
C	1.838001	0.783753	-0.536128
C	0.546427	1.325459	0.191471
C	0.749396	1.535162	1.702675
C	1.273688	-0.166229	-1.653585
C	-0.063923	-0.599585	-1.121399
C	-0.427729	0.206353	-0.128174
O	2.566463	1.877501	-1.066313
O	-1.614707	0.118936	0.622052
C	-3.781867	-1.266233	-0.060021
F	-5.016110	-1.211408	-0.555080
F	-3.811628	-1.748715	1.178013
F	-3.029109	-2.056582	-0.829670
S	-3.078054	0.468445	-0.051271
O	-3.827166	1.222709	0.936647
O	-2.932800	0.891561	-1.433851
C	0.054036	2.643083	-0.443909
C	3.992291	-0.535334	-0.204795
O	4.420358	-0.155886	-1.288950
O	4.645996	-1.445563	0.542183
C	5.898954	-1.904895	0.005564
H	0.481420	-0.482732	2.472593
H	1.539270	0.510583	3.448771
H	3.144474	-0.906534	2.264828
H	1.470194	2.350301	1.834719
H	-0.193955	1.861252	2.152170
H	1.159858	0.411698	-2.579984

H	1.943795	-1.000620	-1.879861
H	-0.656093	-1.411159	-1.525524
H	3.363550	1.488403	-1.466800
H	0.792823	3.432567	-0.293000
H	-0.888093	2.951679	0.021006
H	-0.113709	2.536611	-1.517687
H	6.283925	-2.616747	0.734947
H	5.747298	-2.389110	-0.961724
H	6.592192	-1.070232	-0.121483

HF = -1653.3814056

XII:

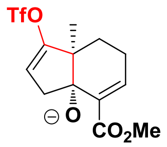

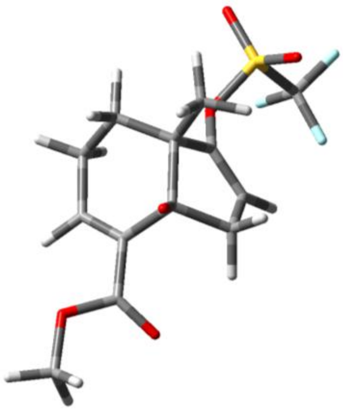

Dihedral angle $\Phi_{((O\text{-})C\text{-}C\text{-}C\text{-}O(Tf))}$ = 156.6°

Atomic Type	Coordinates (Å) X	Y	Z
C	1.270292	1.041539	2.112543
C	2.461782	0.249546	1.648319
C	2.685544	-0.047652	0.355968
C	1.915228	0.511420	-0.878468
C	0.543206	1.290124	-0.325946
C	0.786830	1.993516	1.013755
C	1.232794	-0.704457	-1.673935
C	-0.072897	-0.944752	-0.972910
C	-0.402938	0.136907	-0.269823
O	2.748732	1.266188	-1.548756
O	-1.568802	0.167413	0.603893
C	-3.817536	-1.177344	0.194473
F	-5.115353	-1.081522	-0.125011
F	-3.712305	-1.641400	1.441639
F	-3.233117	-2.039508	-0.642894
S	-3.039336	0.516278	0.056494
O	-3.716445	1.330638	1.057507
O	-3.058367	0.862835	-1.357329

C	0.164655	2.326230	-1.394659
C	3.916569	-0.814507	0.010827
O	4.028224	-1.697527	-0.820087
O	4.989530	-0.464918	0.796715
C	6.177364	-1.209228	0.540714
H	0.449925	0.371023	2.414531
H	1.532404	1.610633	3.015661
H	3.181745	-0.061407	2.404004
H	1.563386	2.748219	0.834008
H	-0.116411	2.526211	1.342961
H	1.071624	-0.338858	-2.697456
H	1.878678	-1.583253	-1.731313
H	-0.667304	-1.852307	-1.022898
H	1.089116	2.847813	-1.667216
H	-0.590458	3.036686	-1.032945
H	-0.227895	1.849264	-2.297582
H	6.933065	-0.812823	1.222858
H	6.027843	-2.278867	0.723919
H	6.502302	-1.085975	-0.496747

HF = -1652.781674

The full citation for reference 49 is given below:

Gaussian 03, Revision E.01, Frisch, M. J.; Trucks, G. W.; Schlegel, H. B.; Scuseria, G. E.; Robb, M. A.; Cheeseman, J. R.; Montgomery, Jr. J. A.; Vreven, T.; Kudin, K. N.; Burant, J. C. ; Millam, J. M.; Iyengar, S. S.; Tomasi, J.; Barone, V.; Mennucci, B.; Cossi, M.; Scalmani, G.; Rega, N.; Petersson, G. A.; Nakatsuji, H.; Hada, M.; Ehara, M.; Toyota, K.; Fukuda, R.; Hasegawa, J.; Ishida, M.; Nakajima, T.; Honda, Y.; Kitao, O.; Nakai, H.; Klene, M.; Li, X.; Knox, J. E.; Hratchian, H. P.; Cross, J. B.; Bakken, V.; Adamo, C.; Jaramillo, J.; Gomperts, R.; Stratmann, R. E.; Yazyev, O.; Austin, A. J.; Cammi, R.; Pomelli, C.; Ochterski, J. W.; Ayala, P. Y.; Morokuma, K.; Voth, G. A.; Salvador, P.; Dannenberg, J. J.; Zakrzewski, V. G.; Dapprich, S.; Daniels, A. D.; Strain, M. C.; Farkas, O.; Malick, D. K.; Rabuck, A. D.; Raghavachari, K.; Foresman, J. B.; Ortiz, J. V.; Cui, Q.; Baboul, A. G.; Clifford, S.; Cioslowski, J.; Stefanov, B. B.; Liu, G.; Liashenko, A.; Piskorz, P.; Komaromi, I.; Martin, R. L.; Fox, D. J.; Keith, T.; Al-Laham, M. A.; Peng, C. Y.; Nanayakkara, A.; Challacombe, M.; Gill, P. M. W.; Johnson, B.; Chen, W.; Wong, M. W.; Gonzalez, C.; Pople, J. A. Gaussian, Inc., Wallingford CT, 2004.

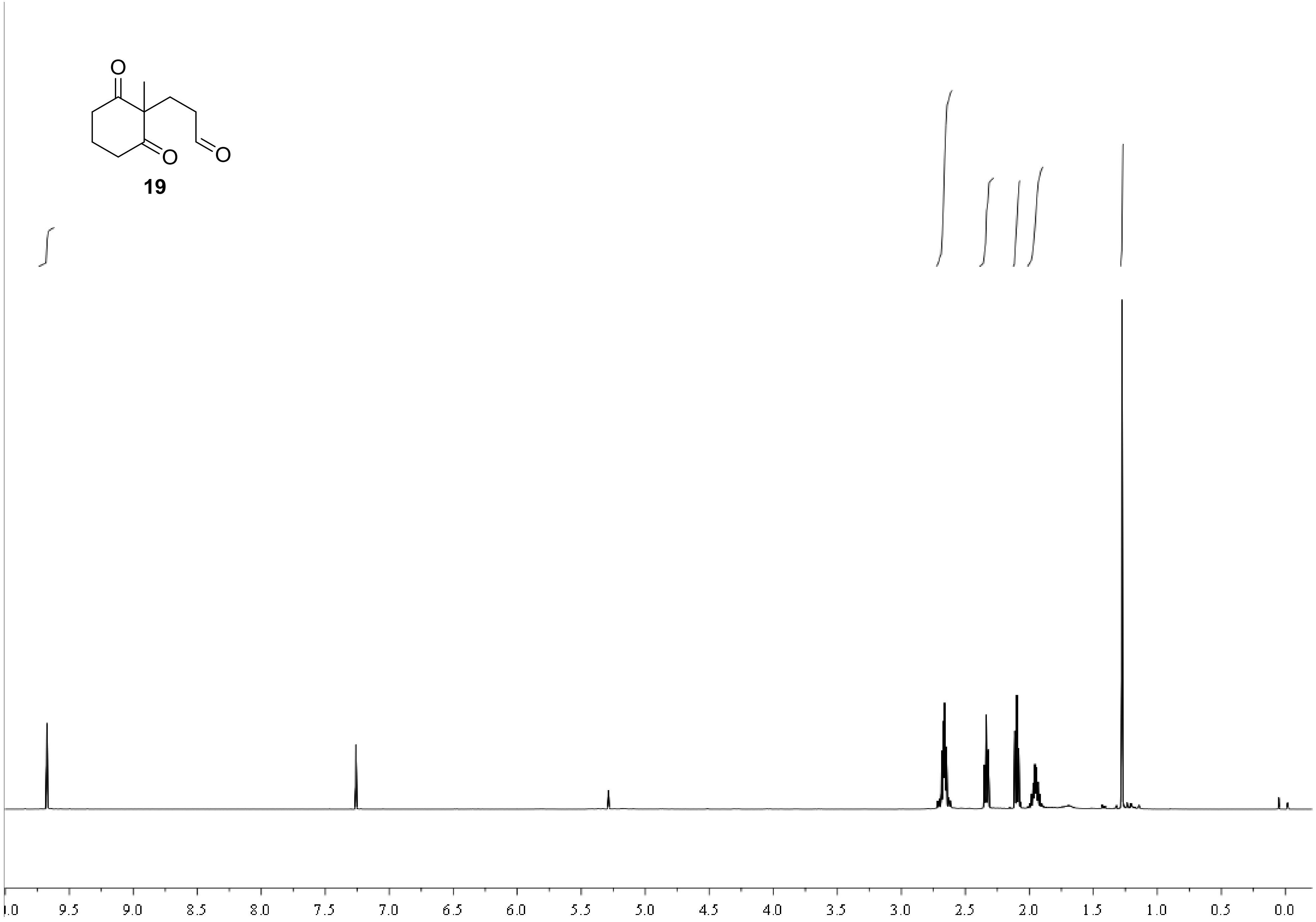
19
.0
9.5
9.0
8.5
8.0
7.5
7.0
6.5
6.0
5.5
5.0
4.5
4.0
3.5
3.0
2.5
2.0
1.5
1.0
0.5
0.0

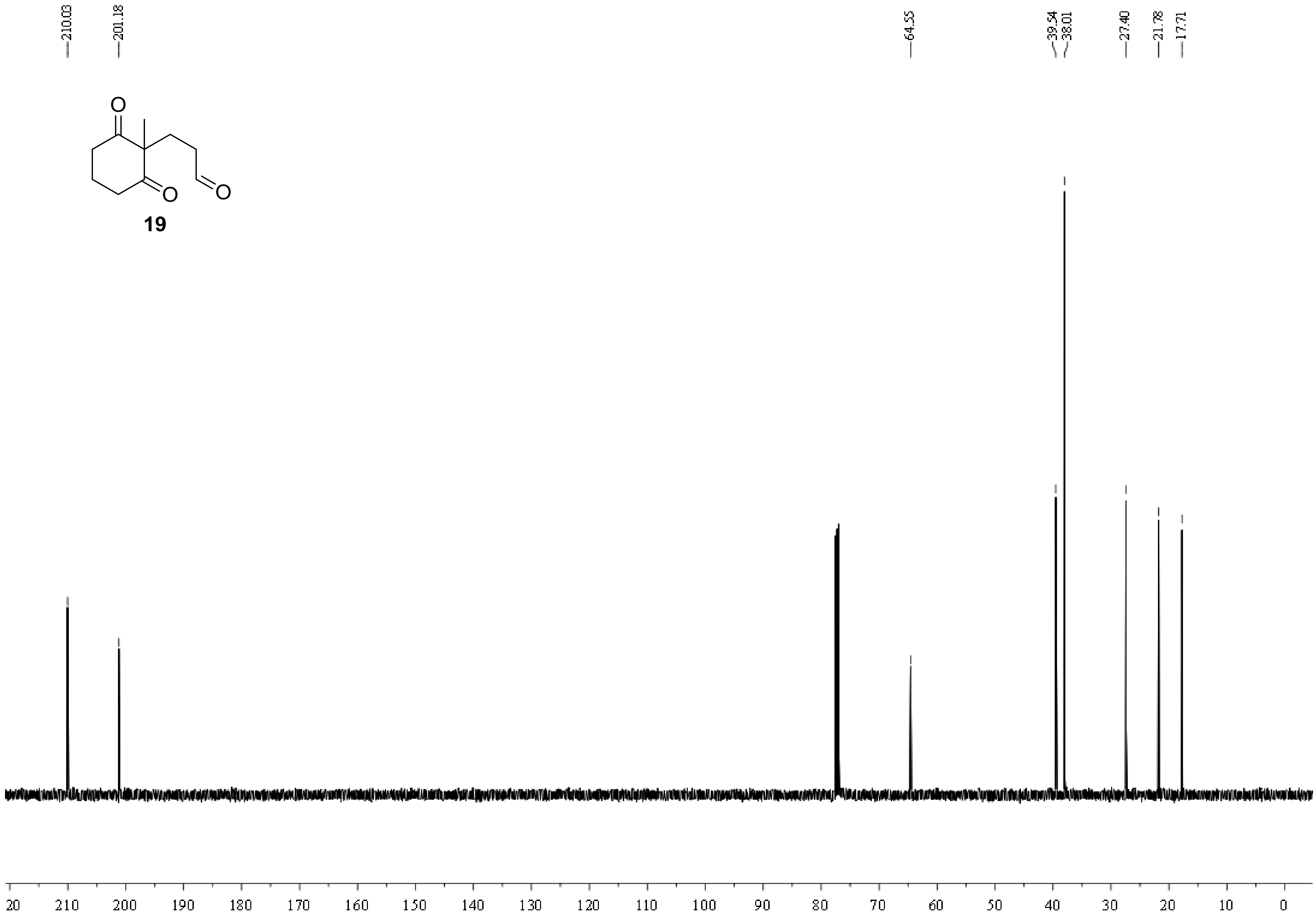
210.03
201.18
64.55
39.54
38.01
27.40
21.78
17.71
O
O
O
19
20
210
200
190
180
170
160
150
140
130
120
110
100
90
80
70
60
50
40
30
20
10
0

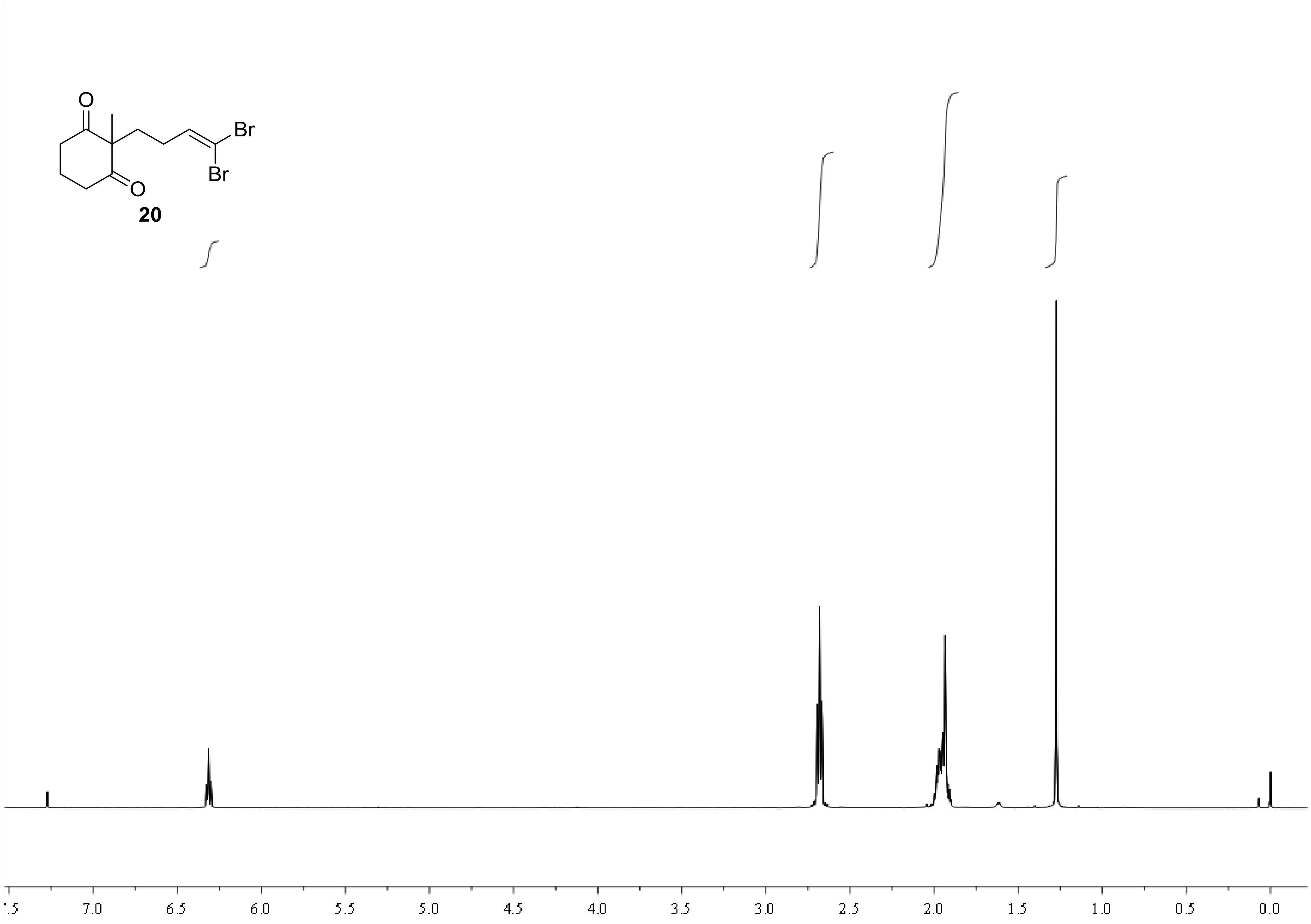
O
Br
Br
O
20
7.0
6.5
6.0
5.5
5.0
4.5
4.0
3.5
3.0
2.5
2.0
1.5
1.0
0.5
0.0

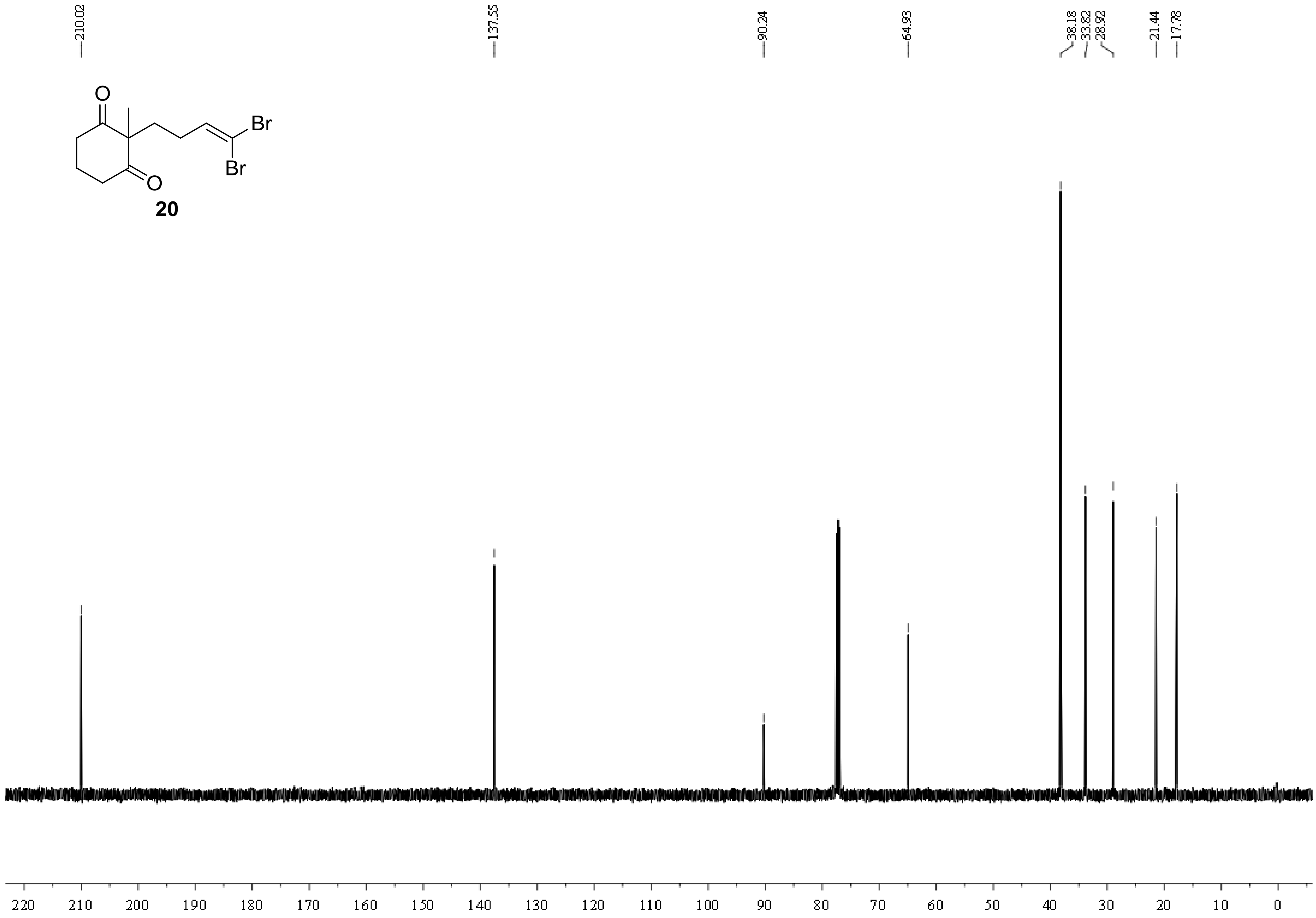
210.02
137.55
90.24
64.93
38.18
33.82
28.92
21.44
17.78
O
Br
Br
O
20
220 210 200 190 180 170 160 150 140 130 120 110 100 90 80 70 60 50 40 30 20 10 0

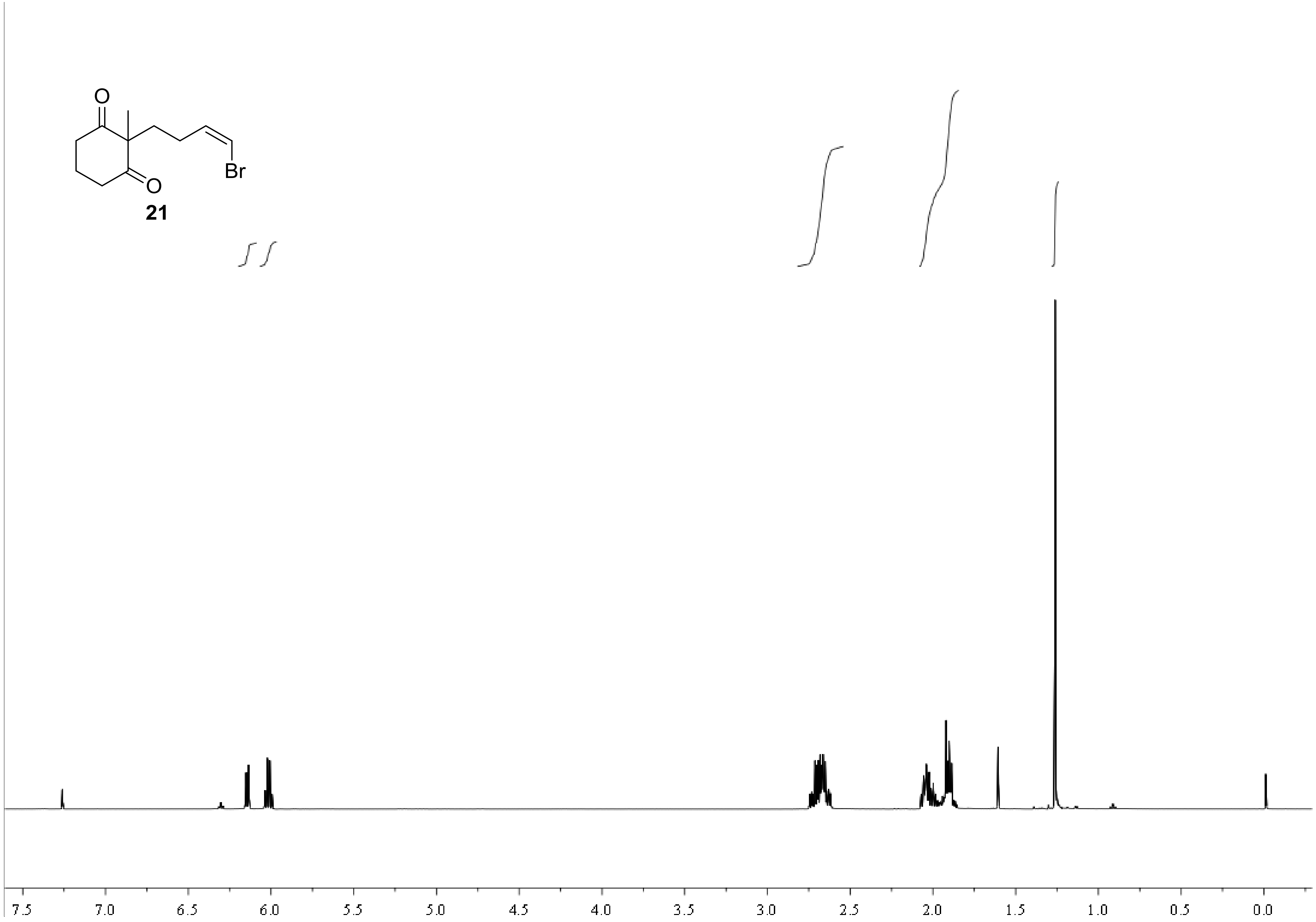
Br
21
7.5
7.0
6.5
6.0
5.5
5.0
4.5
4.0
3.5
3.0
2.5
2.0
1.5
1.0
0.5
0.0

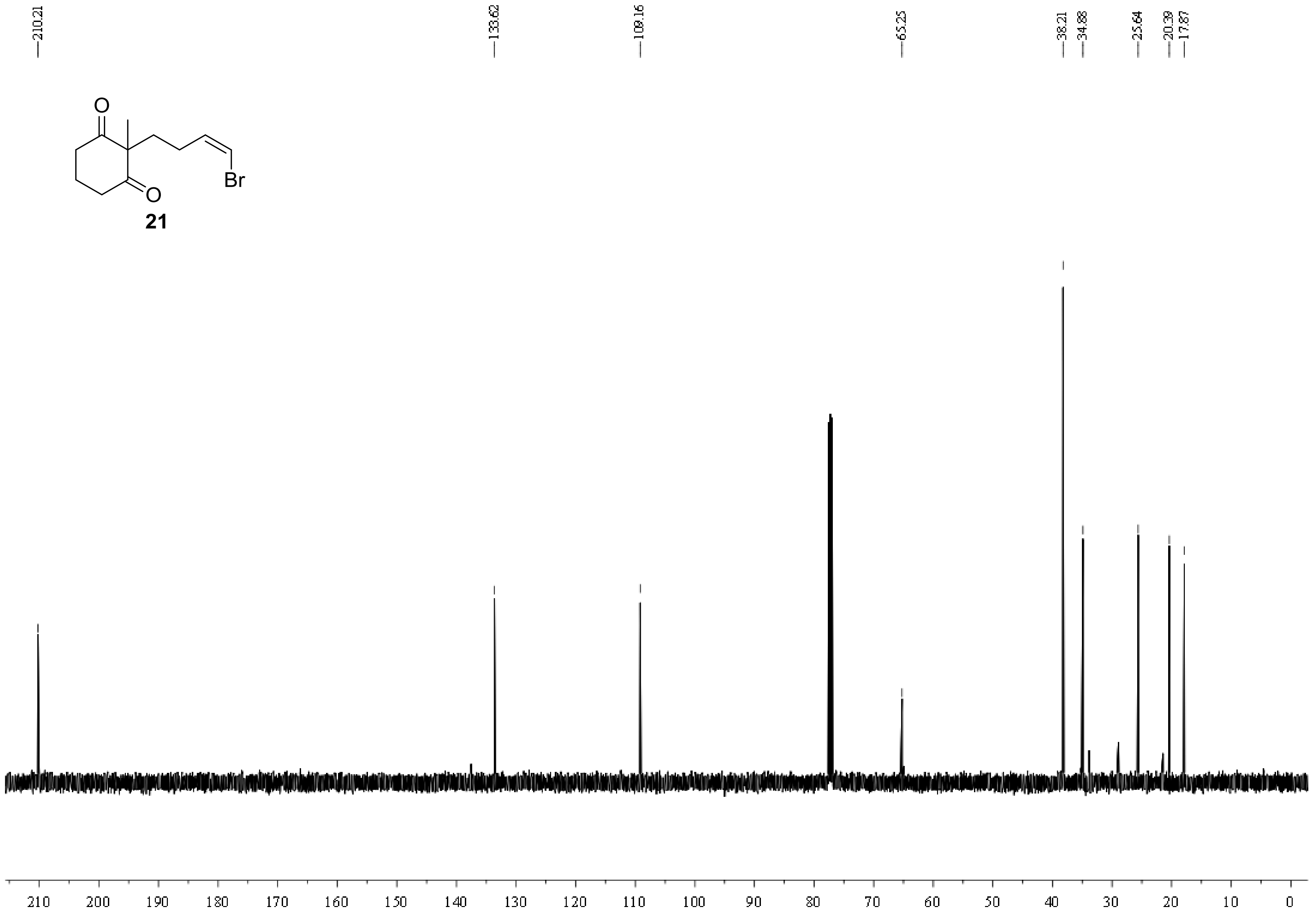
210.21
133.62
109.16
65.25
38.21
34.88
25.64
20.39
17.87
O
O
Br
21
210 200 190 180 170 160 150 140 130 120 110 100 90 80 70 60 50 40 30 20 10 0

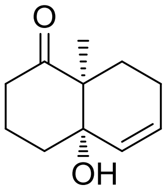

22

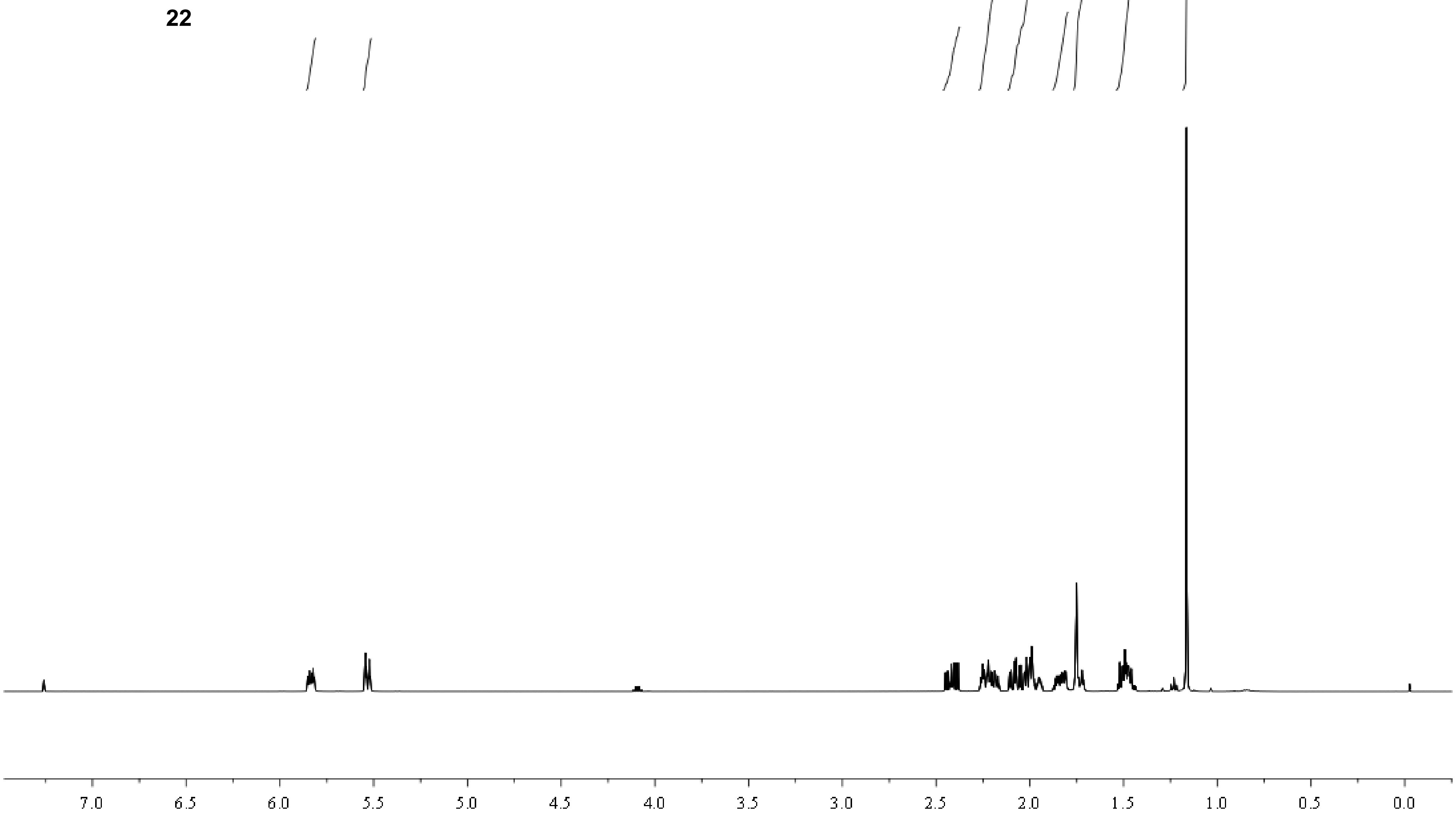

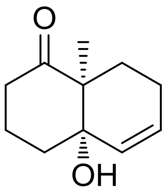
O
OH

22

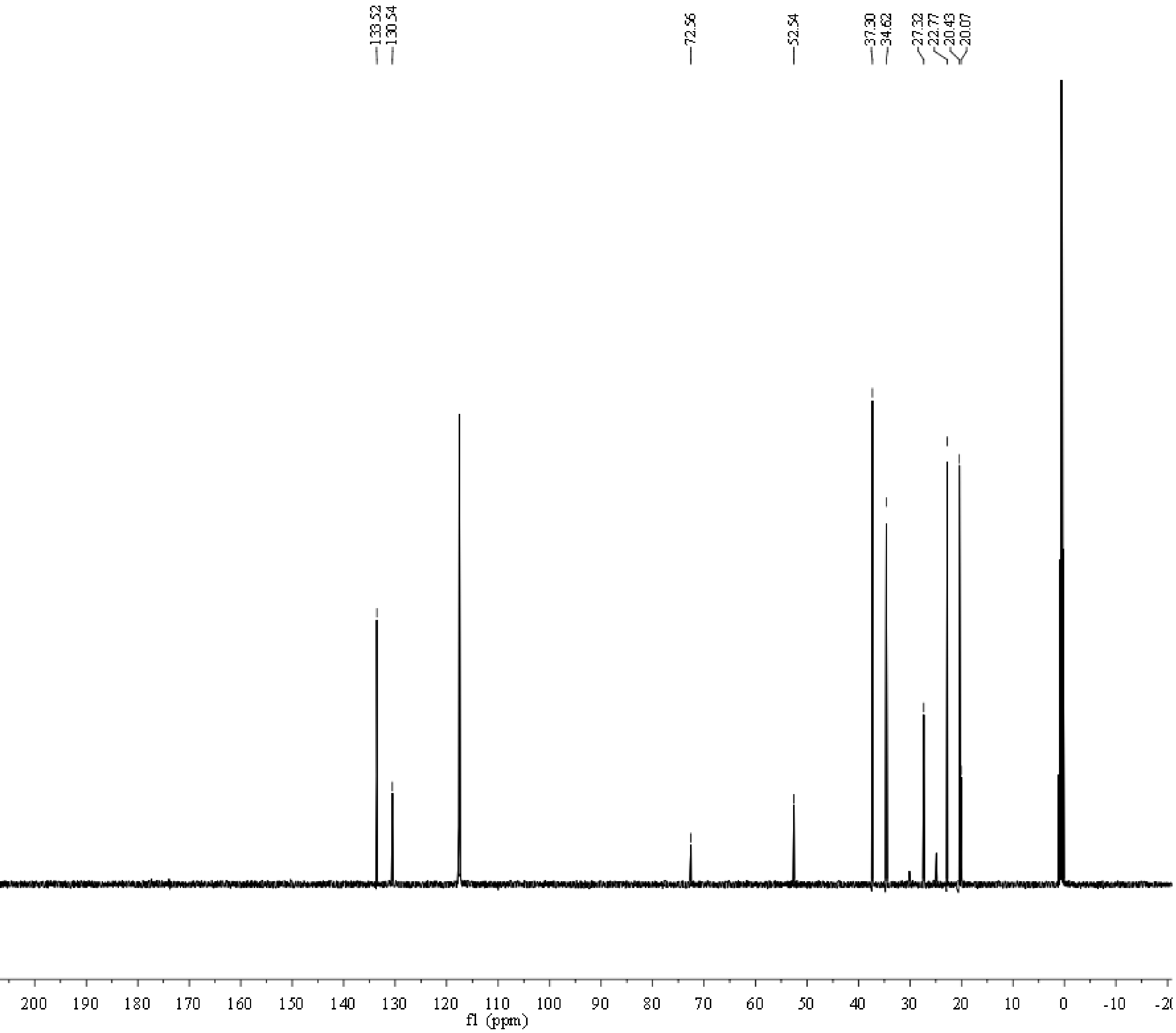
213.76
133.52
130.54
72.56
52.54
37.30
34.62
27.32
22.77
20.43
20.07
40 230 220 210 200 190 180 170 160 150 140 130 120 110 100 90 80 70 60 50 40 30 20 10 0 -10 -20
f1 (ppm)

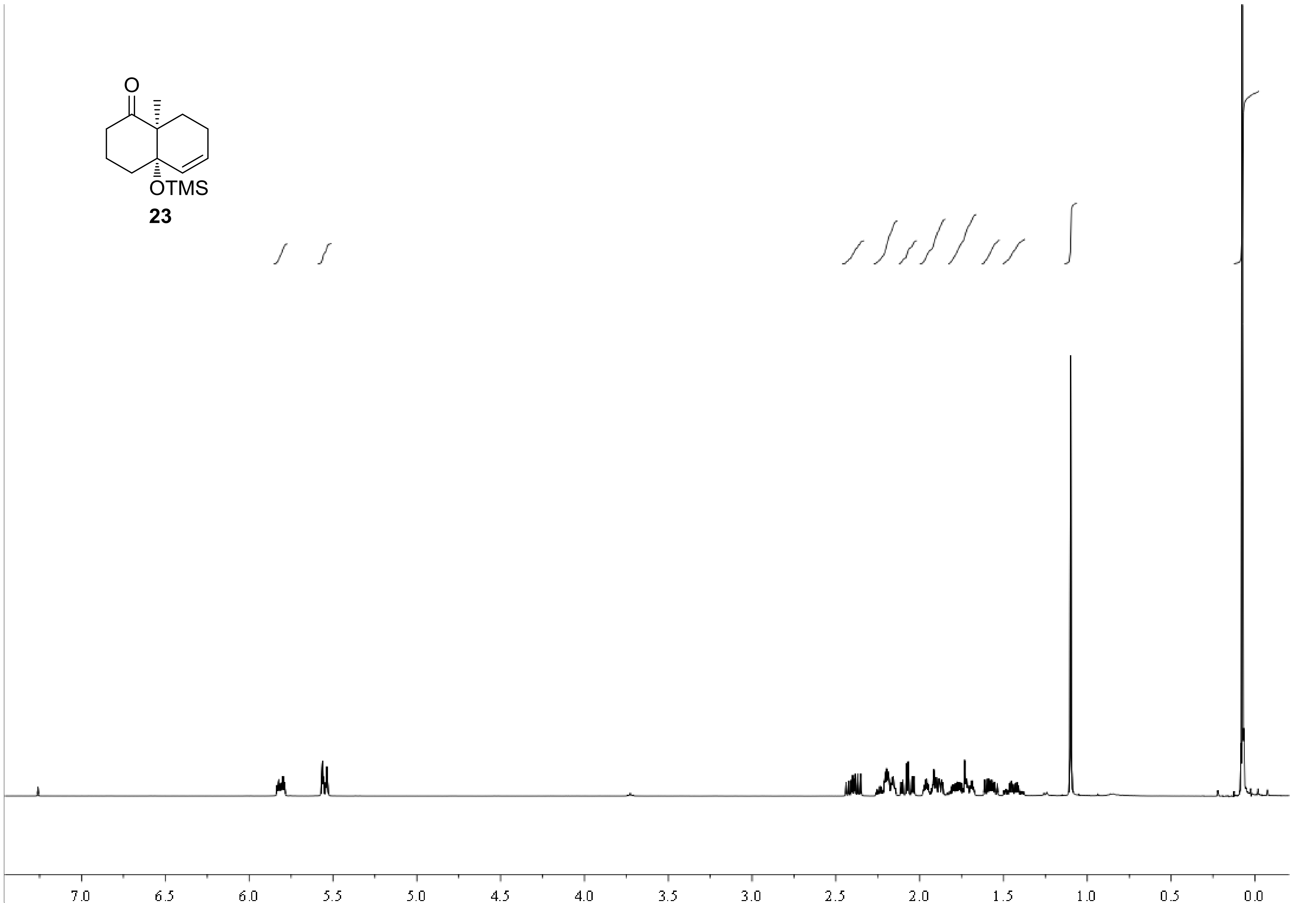
O
OTMS
23
7.0
6.5
6.0
5.5
5.0
4.5
4.0
3.5
3.0
2.5
2.0
1.5
1.0
0.5
0.0

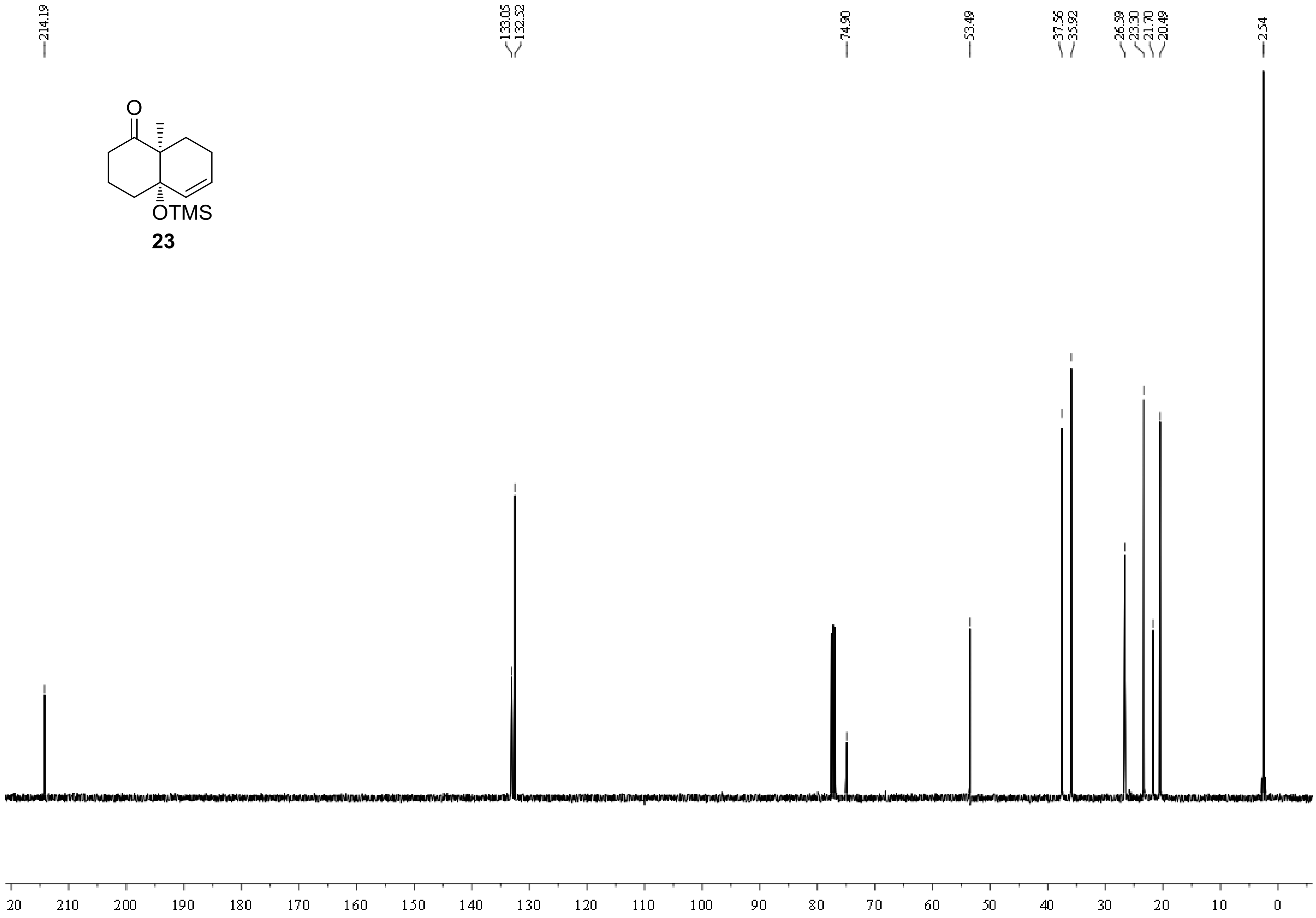
214.19
133.05
132.52
74.90
53.49
37.56
35.92
26.59
23.30
21.70
20.49
2.54
O
OTMS
23
20 210 200 190 180 170 160 150 140 130 120 110 100 90 80 70 60 50 40 30 20 10 0

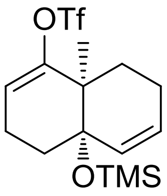

24

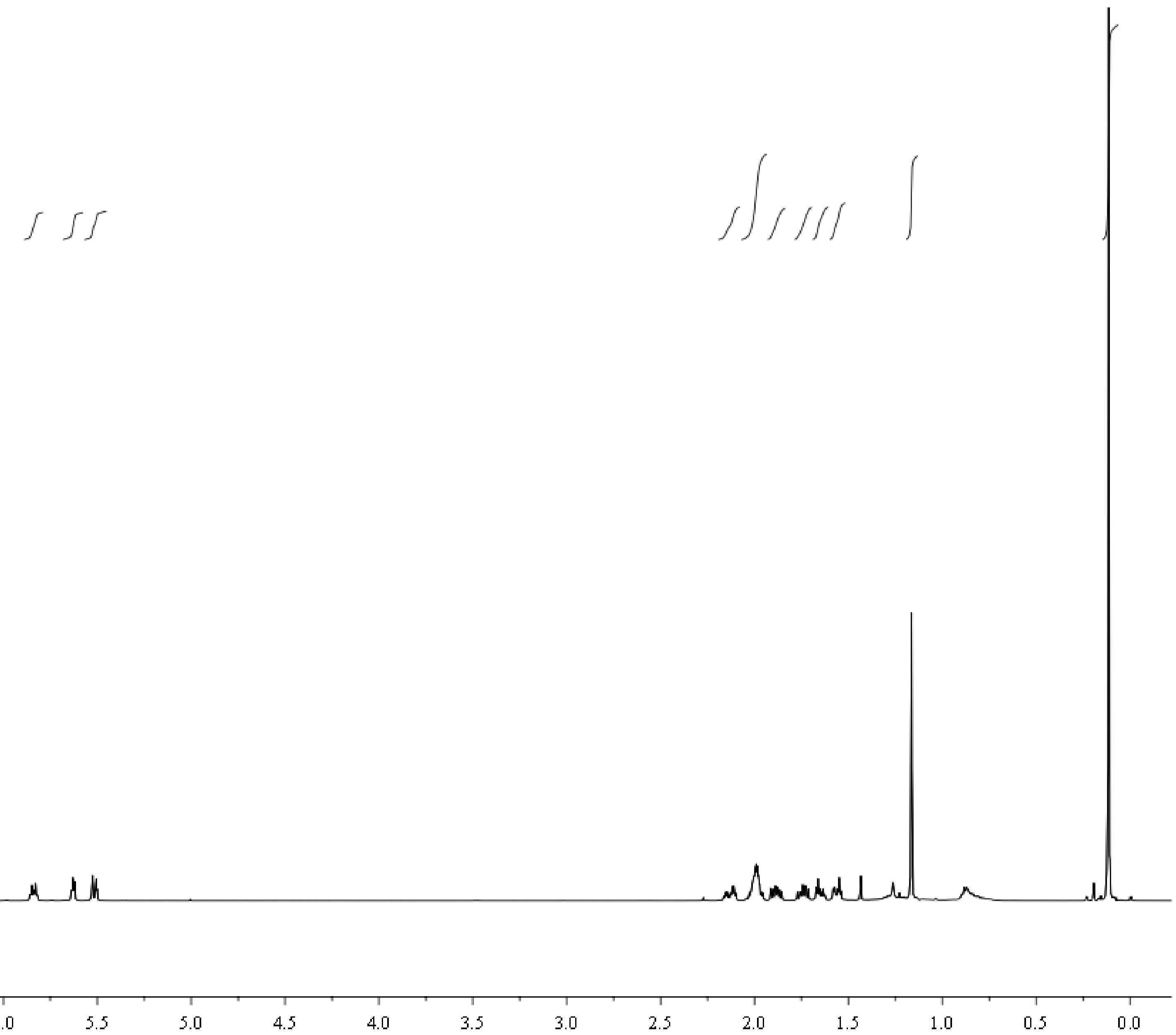

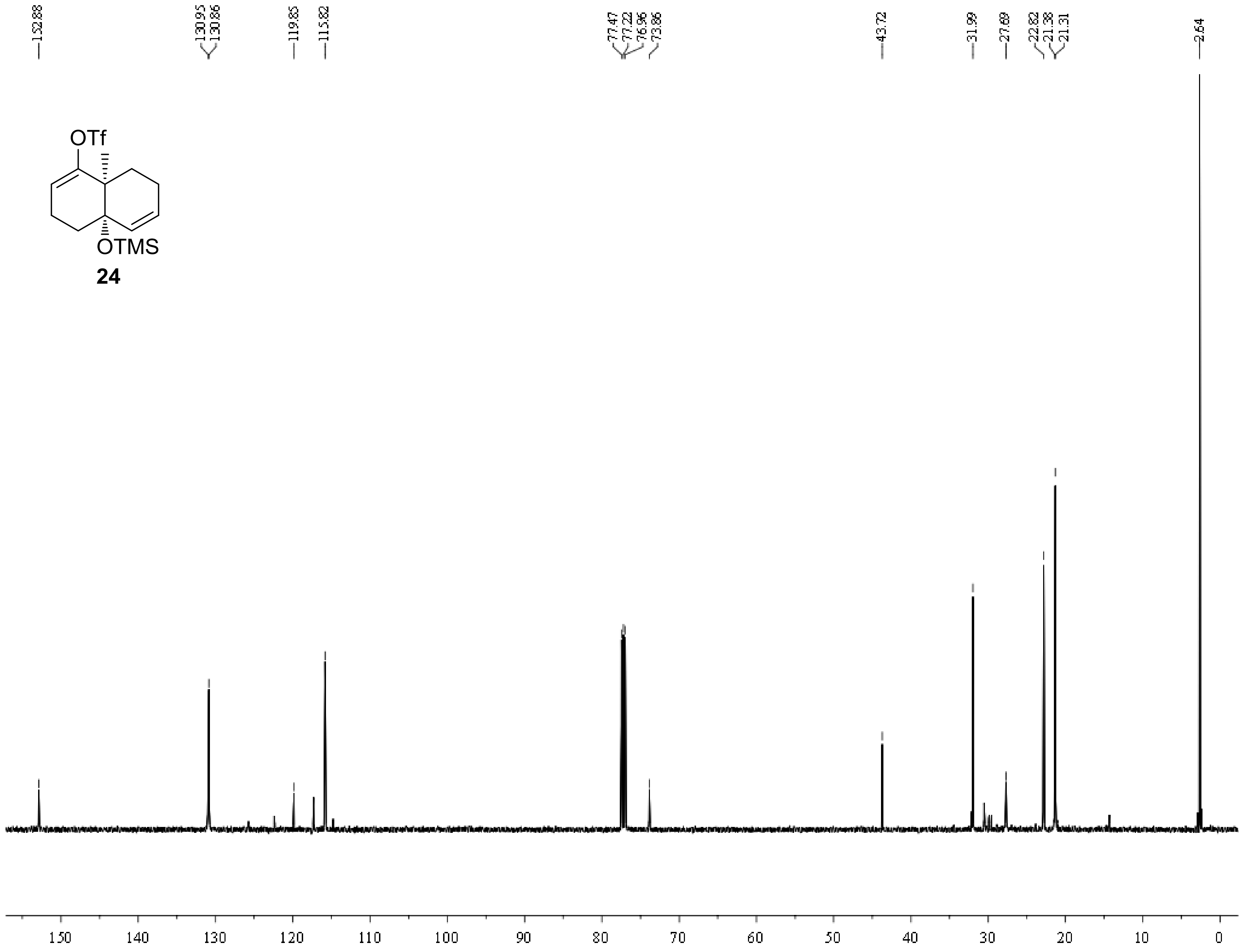
152.88
130.95
130.86
119.85
115.82
77.47
77.22
76.96
73.86
43.72
31.99
27.69
22.82
21.38
21.31
2.64
OTf
OTMS
24
150
140
130
120
110
100
90
80
70
60
50
40
30
20
10
0

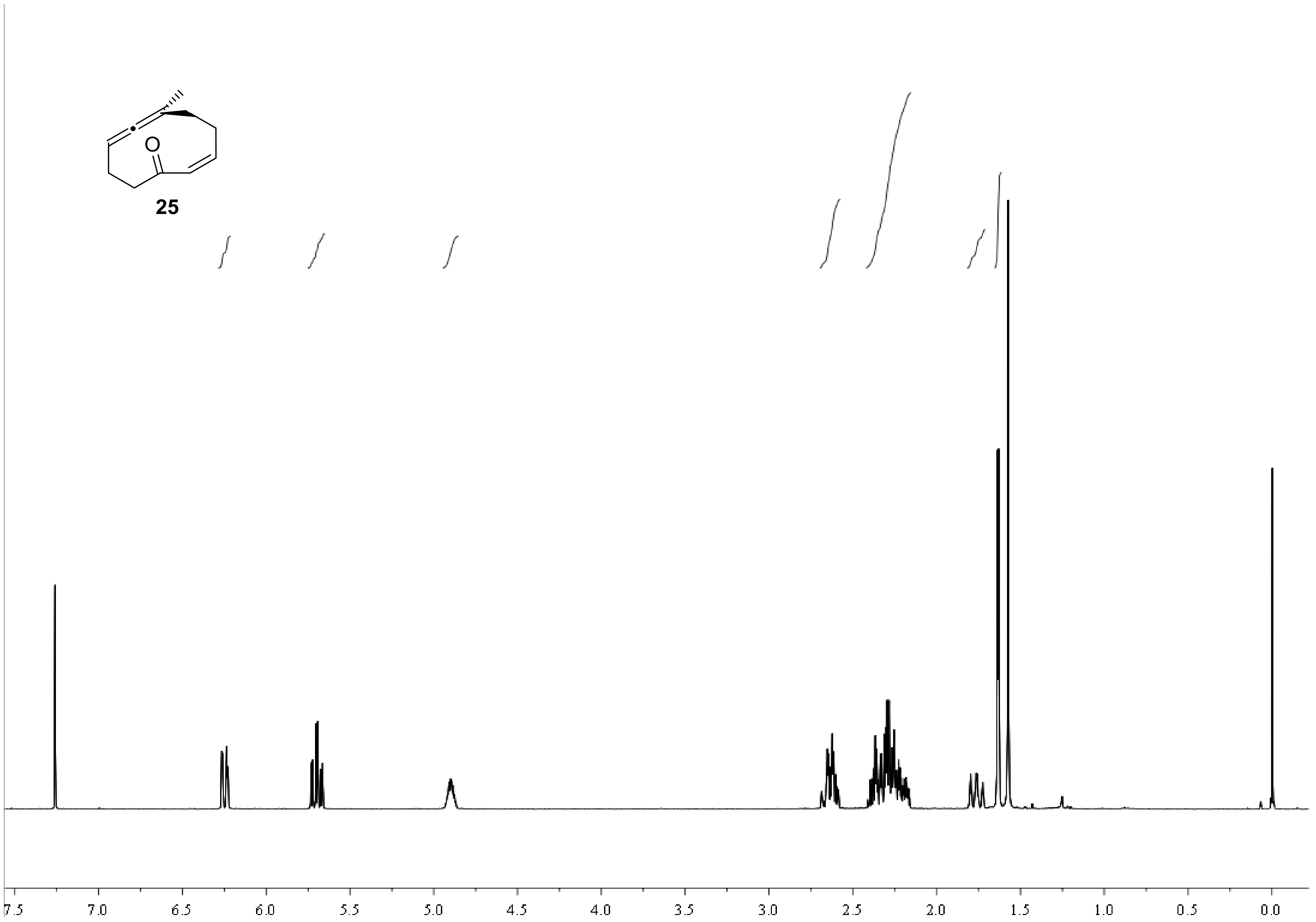
O
25
7.5
7.0
6.5
6.0
5.5
5.0
4.5
4.0
3.5
3.0
2.5
2.0
1.5
1.0
0.5
0.0

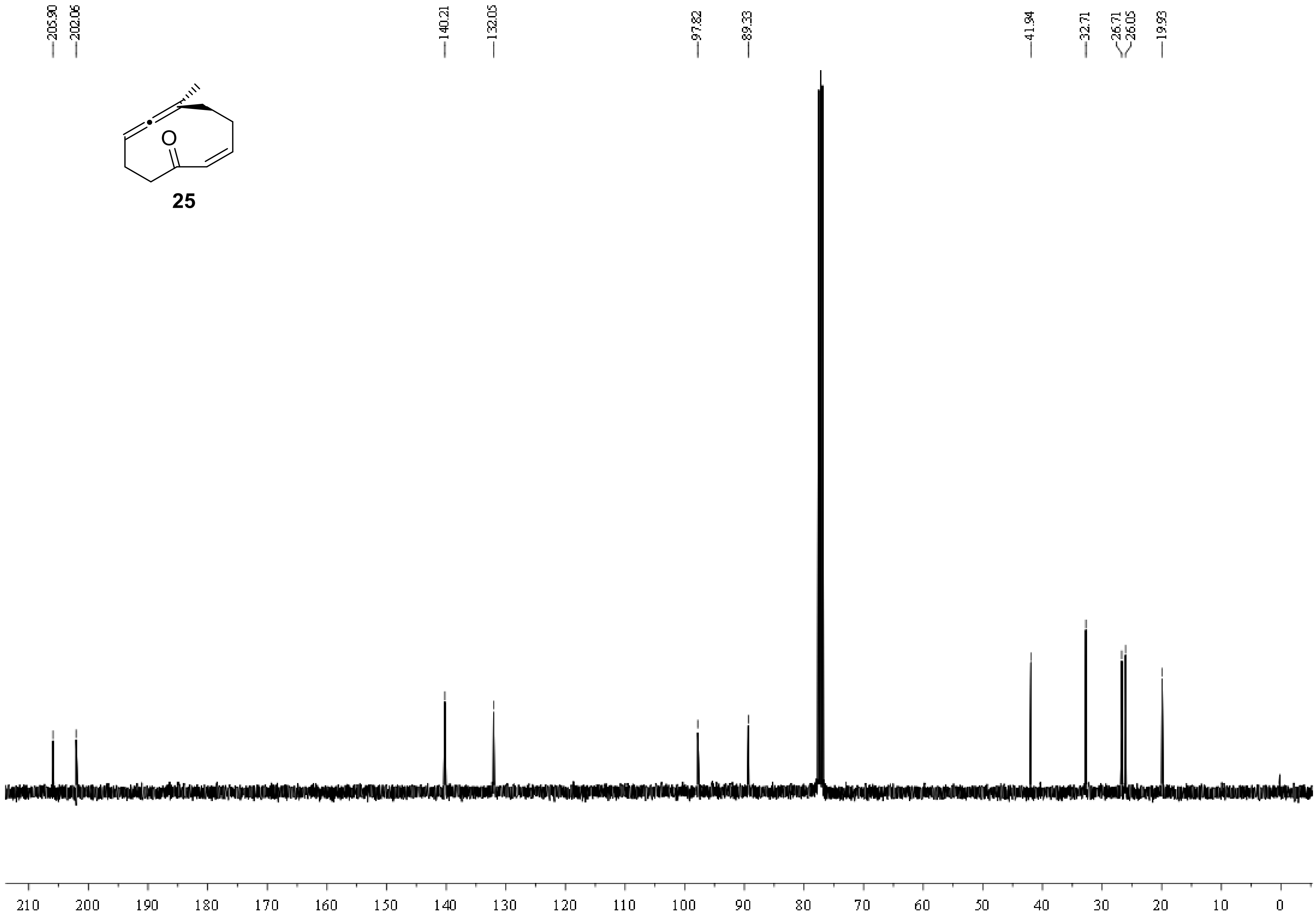
205.90
202.06
140.21
132.05
97.82
89.33
41.94
32.71
26.71
26.05
19.93
O
25
210 200 190 180 170 160 150 140 130 120 110 100 90 80 70 60 50 40 30 20 10 0

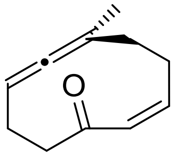

25

Gcosy

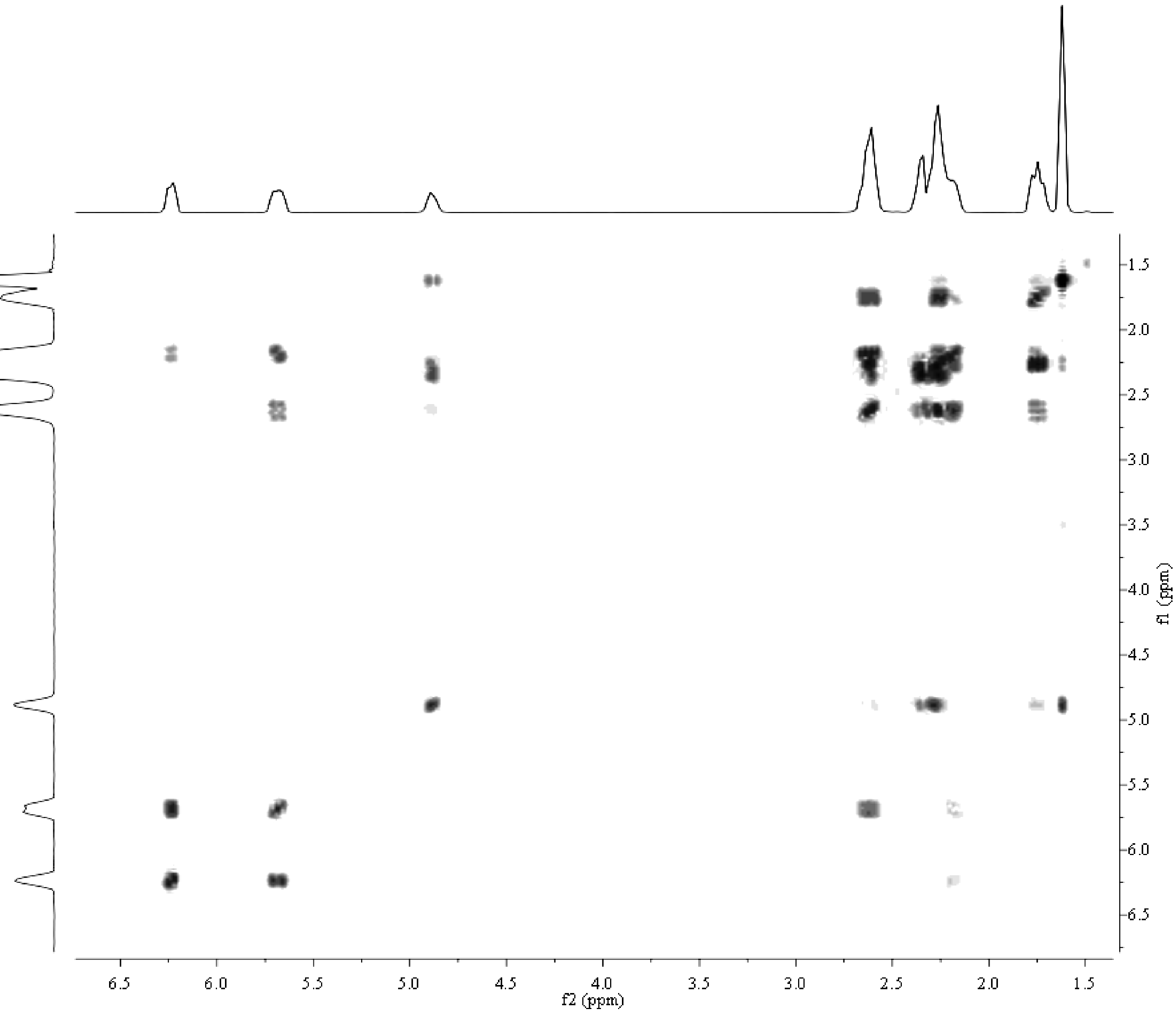

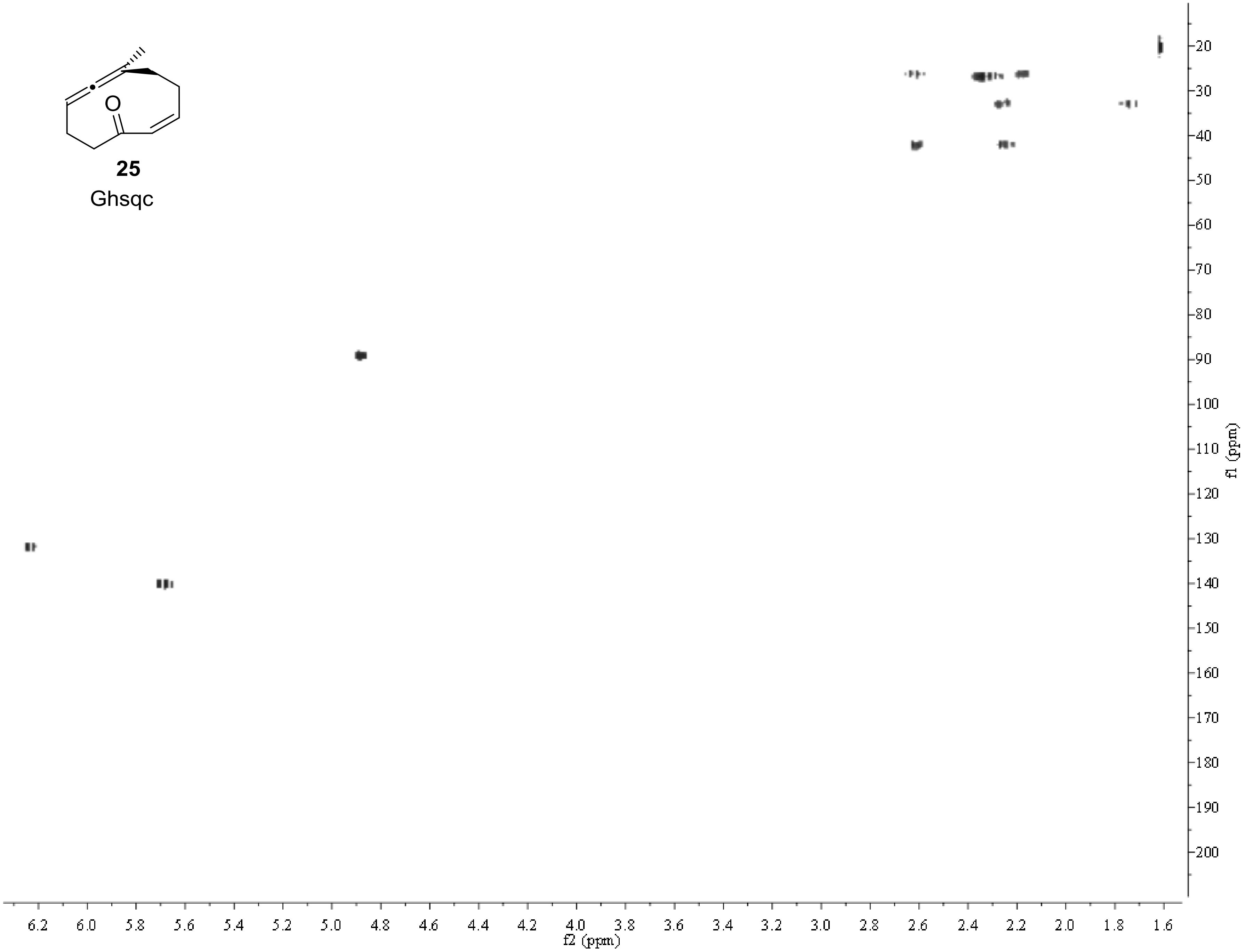

25

Ghsqc
